# Supplementary material for: Sirt6 deficiency exacerbates podocyte injury and proteinuria through targeting Notch signaling
Source: Nat Commun. 2017 Sep 4;8:413. doi: 10.1038/s41467-017-00498-4 (PMC5583183; doi:10.1038/s41467-017-00498-4)
Supplement: Supplementary file 1 — Supplementary Information [file 41467_2017_498_MOESM1_ESM.pdf]

Description of Supplementary Files

File name: Supplementary Information
Description: Supplementary figures and supplementary tables.

File name: Peer review file

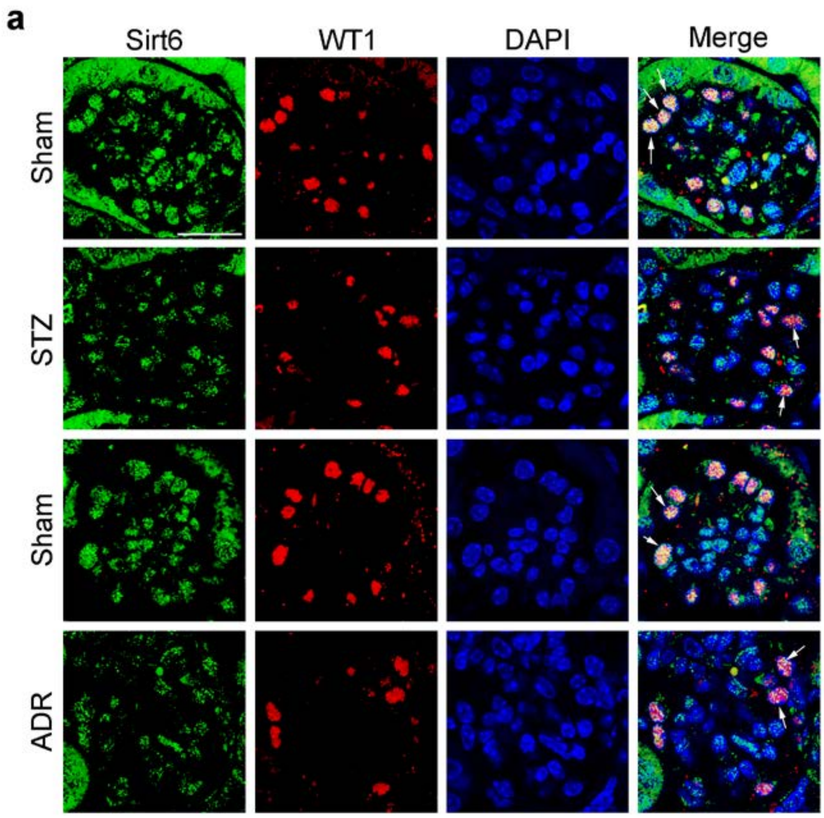

Supplementary Figure 1. The expression levels of Sirt6 in the kidney from STZ-induced diabetic mice and ADR-treated mice. Representative confocal microscopic images showing the expression of Sirt6 in podocytes from STZ-induced diabetic mice and ADR-treated mice, WT1 were used as podocyte marker. The arrows indicate representative podocytes. Scale bar, 25 μm.

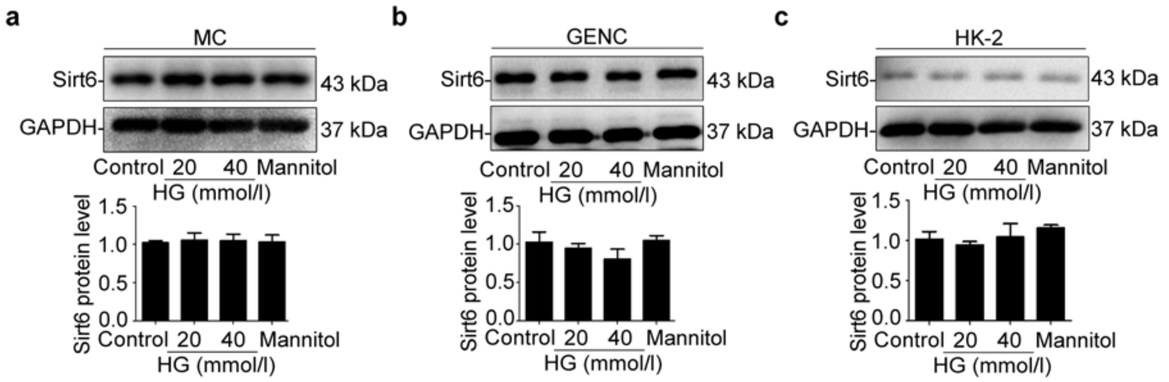

Supplementary Figure 2. The expression levels of Sirt6 in different renal cells with HG treatment. (**a)** Representative Western blot gel documents and summarized data showing that the expression level of Sirt6 was not changed in rat glomerular mesangial cells (MC) under HG (HG, final concentration 20 or 40 mmol/l in medium) condition for 24 hours. (**b)** Representative Western blot gel documents and summarized data showing a decrease tendency of Sirt6 expression in rat glomerular endothelial cells (GENC) under HG (HG, final concentration 20 or 40 mmol/l in medium) condition for 24 hours. (**c)** Representative Western blot gel documents and summarized data showing that the expression level of Sirt6 was not changed in human tubule epithelial cells (HK-2) treated with HG (HG, final concentration 20 or 40 mmol/l in medium) for 24 hours, (n=6). Data are expressed as means ± SE.

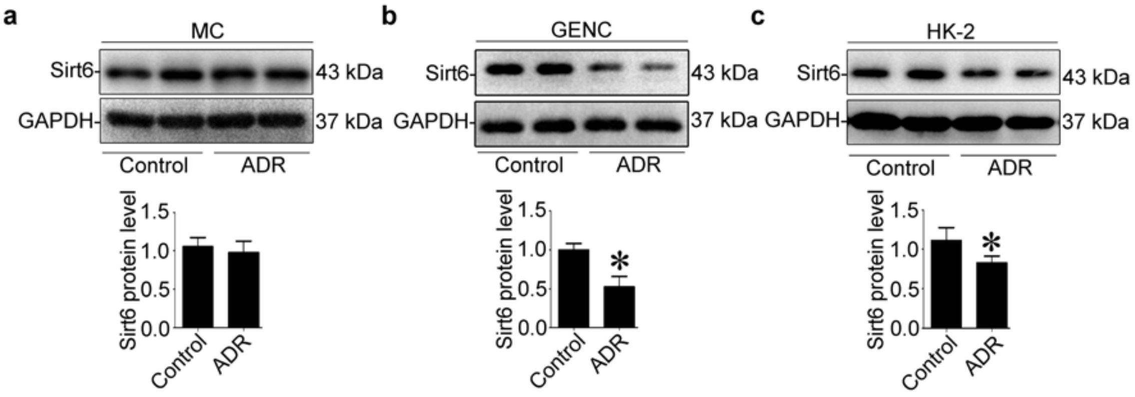

Supplementary Figure 3. The expression levels of Sirt6 in different renal cells with ADR treatment. (**a)** Representative Western blot gel documents and summarized data showing that the expression level of Sirt6 was not changed in rat glomerular mesangial cells (MC) under ADR (ADR, 0.4 μg/ml) condition for 24 hours. (**b)** Representative Western blot gel documents and summarized data showing that Sirt6 was reduced in rat glomerular endothelial cells (GENC) under ADR (ADR, 0.4μg/ml) condition for 24 hours. (**c)** Representative Western blot gel documents and summarized data showing that Sirt6 was reduced in human tubule epithelial cells (HK-2) treated with ADR for 24 hours. *P<0.05 vs. control. (n=6). Data are expressed as means ± SE. Student's t-test was employed for comparisons between two groups.

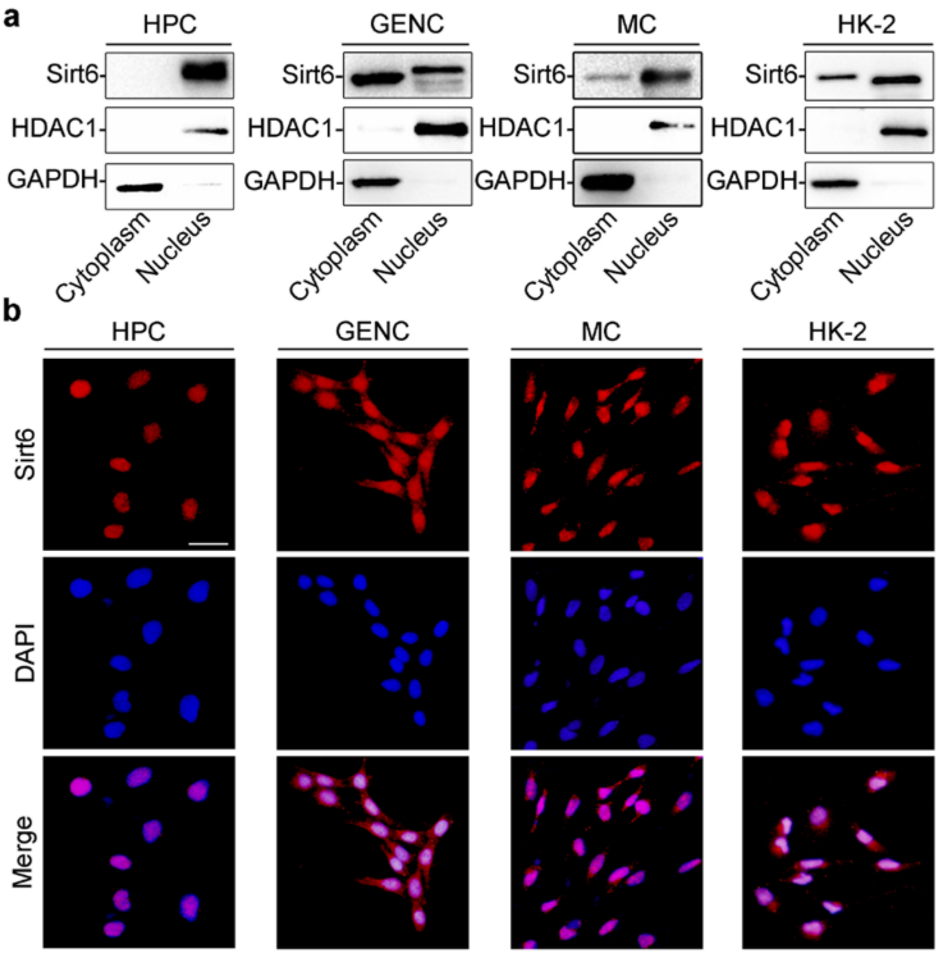

Supplementary Figure 4. Nuclear isolation assay and immunofluorescence staining was used to detect the intracellular localization of Sirt6 in various types of renal cells. In this study, anti-Sirt6 antibody was obtained from Abcam (cat No. AB62739; Lot No.GR285974). **(a)** Representative Western blot gel documents and summarized data showing the intracellular localization of Sirt6 in various types of renal cells including human podocytes (HPC), rat glomerular endothelial cells (GENC), rat glomerular mesangial cells (MC), and human proximal tubule epithelial cells (HK-2). **(b)** Representative confocal microscopic images showing the intracellular localization of Sirt6 in various types of renal cells. Scale bar, 40 μm.

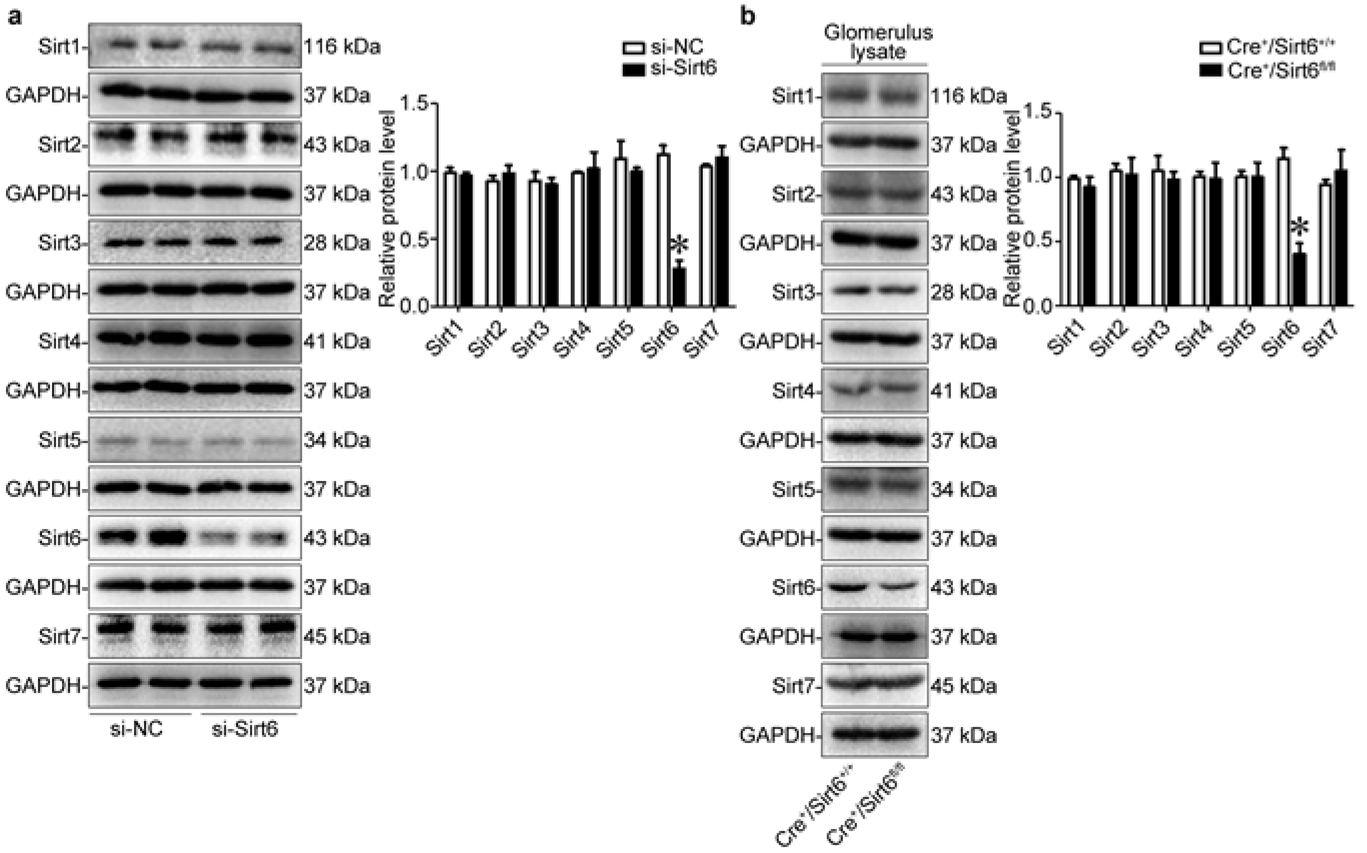

Supplemental Figure 5. The expression levels of SIRTs in *Sirt6*-deficient podocytes or in isolated glomeruli from *Cre*$^{+}$*/Sirt6*$^{+/+}$ mice and *Cre*$^{+}$*/Sirt6*$^{fl/fl}$ mice. (**a**) Representative Western blot gel documents and summarized data showing the expression levels of SIRTs in Sirt6-deficient (si-Sirt6) podocytes. **P*<0.05 vs. si-negative control. (n=6). (**b**) Representative Western blot gel documents and summarized data showing the expression levels of SIRTs in isolated glomeruli from *Cre*$^{+}$*/Sirt6*$^{+/+}$ mice and *Cre*$^{+}$*/Sirt6*$^{fl/fl}$ mice. **P*<0.05 vs. control (*Cre*$^{+}$*/Sirt6*$^{+/+}$ mice). (n=8). Data are expressed as means ± SE. Student's t-test was employed for comparisons between two groups.

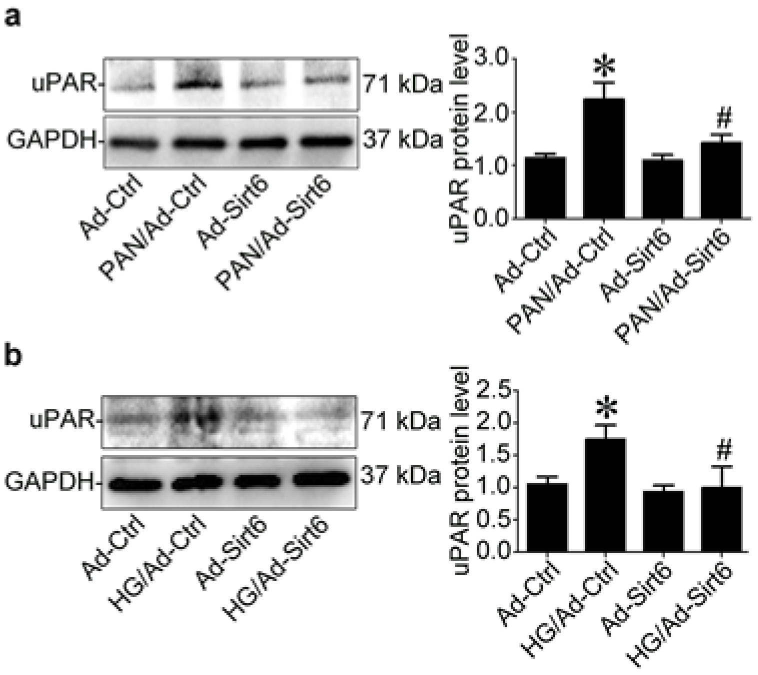

Supplementary Figure 6. The expression levels of urokinase plasminogen activator receptor (uPAR) in podocytes with puromycin aminonucleoside (PAN) or HG treatment, PAN as a positive control. (**a**) Representative Western blot gel documents and summarized data showing the expression level of uPAR in podocytes with PAN (PAN, final concentration 100 µg/ml in medium) treatment for 24 hours. (**b**) Representative Western blot gel documents and summarized data showing the expression level of uPAR in podocytes with HG (HG, final concentration 40 mmol/l in medium) treatment for 24 hours. *P<0.05 vs. control, $^{\#}P$ < 0.05 vs. scramble of PAN or HG treatment. (n=6). Data are expressed as means ± SE. One-way ANOVA followed by Tukey's post-test for multiple comparisons was used for groups of three or more.

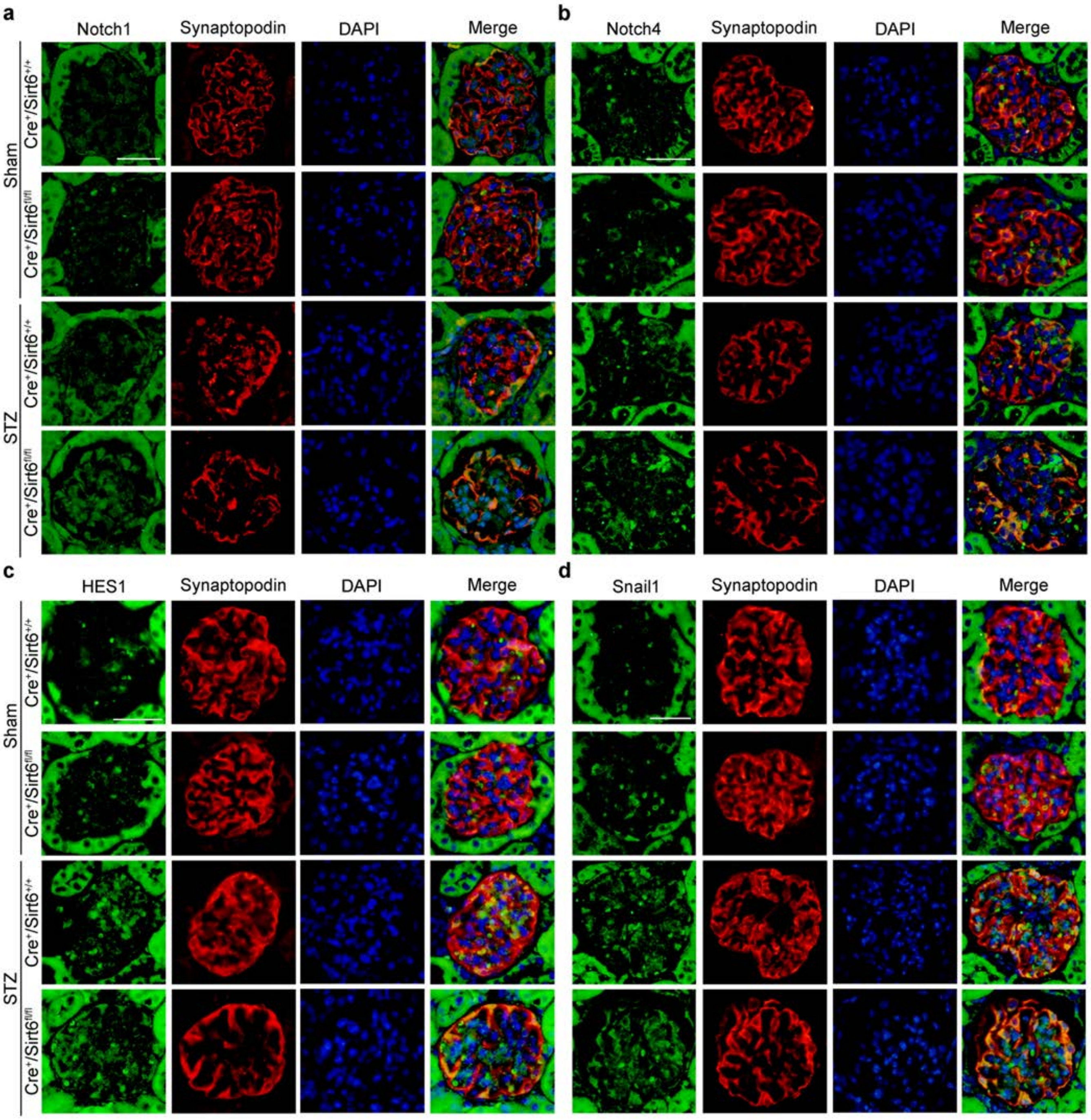

Supplementary Figure 7. Immunofluorescent staining showing more significant increases in the levels of Notch1 and Notch4, as well as HES1 and Snail1 in podocytes from diabetic *Cre⁺/Sirt6^fl/fl^* mice as compared with diabetic *Cre⁺/Sirt6^+/+^* mice. Synaptopodin was used as podocyte marker. Scale bar, 25 μm.

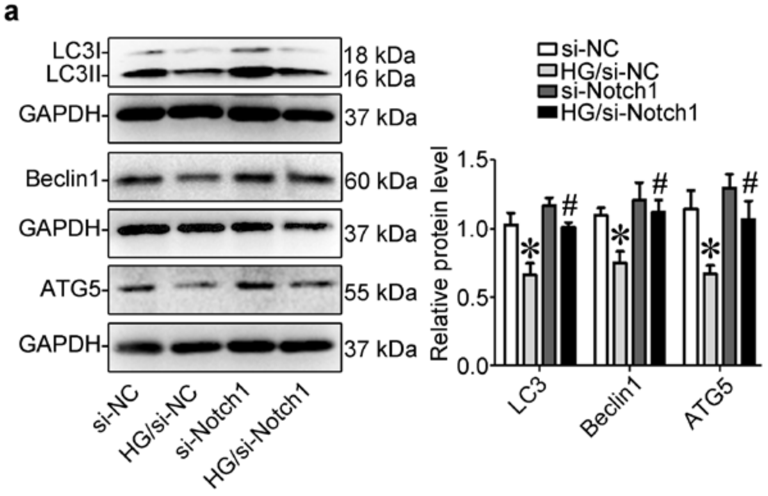

Supplementary Figure 8. Gene silencing of *Notch 1* restored the expression levels of autophagy-associated proteins in podocytes with HG treatment. **P*<0.05 vs. control, #*P* < 0.05 vs. scramble of HG treatment. (n=6). Data are expressed as means ± SE. One-way ANOVA followed by Tukey's post-test for multiple comparisons was used for groups of three or more.

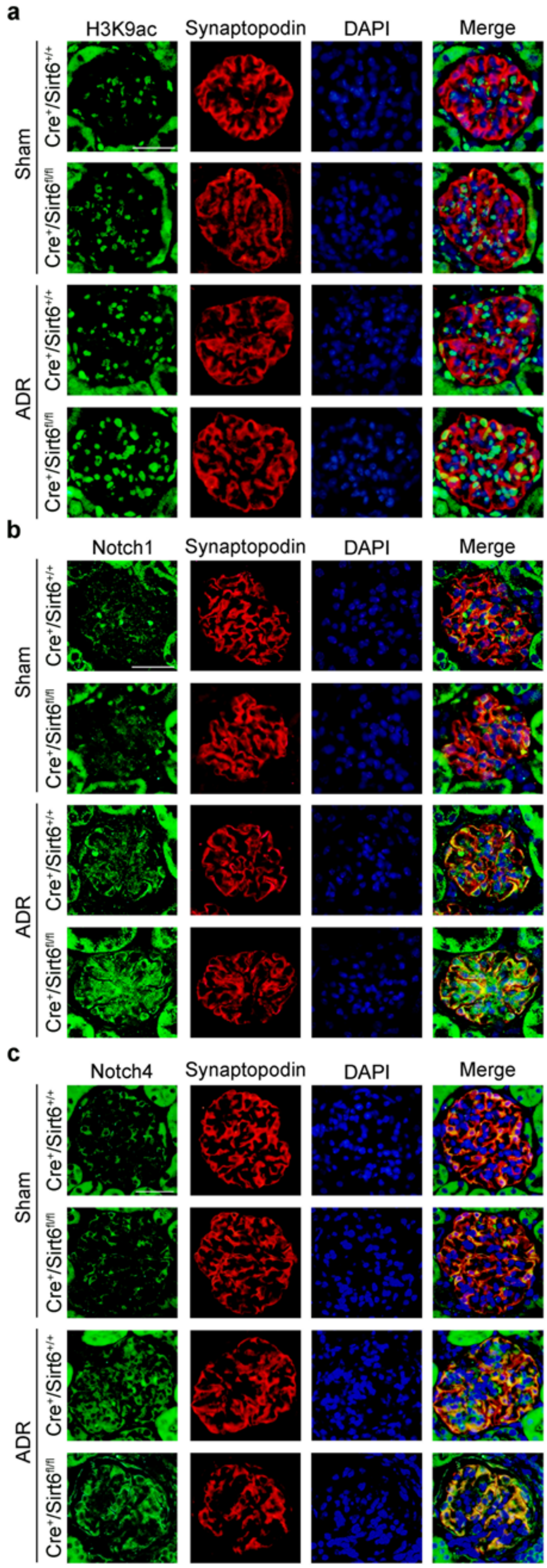

Supplementary Figure 9. Significant increases in the expression levels of H3K9ac, Notch1 and Notch 4 in podocytes from ADR *Cre^{+}/Sirt6$^{fl/fl}$* mice **(a)** Representative confocal microscopic images showing the levels of H3K9ac in podocytes from different groups of mice. **(b)** Representative confocal microscopic images showing the expression level of Notch1 in podocytes from different group of mice. **(c)** Representative confocal microscopic images showing the expression level of Notch4 in podocytes from different group of mice, synaptopodin was used as a podocyte marker. Scale bar, 25 µm.

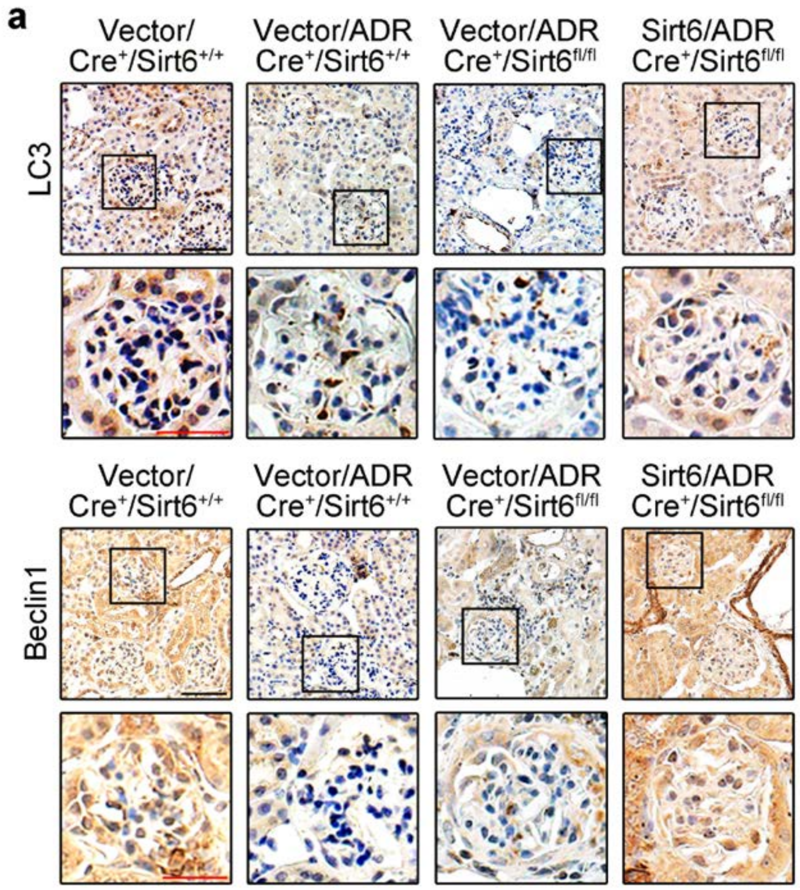

Supplementary Figure 10: Representative photomicrographs of LC3 and Beclin1 immunohistochemical staining in the kidney from different groups of mice. Scale bar: black 50 μm, red 25 μm.

.

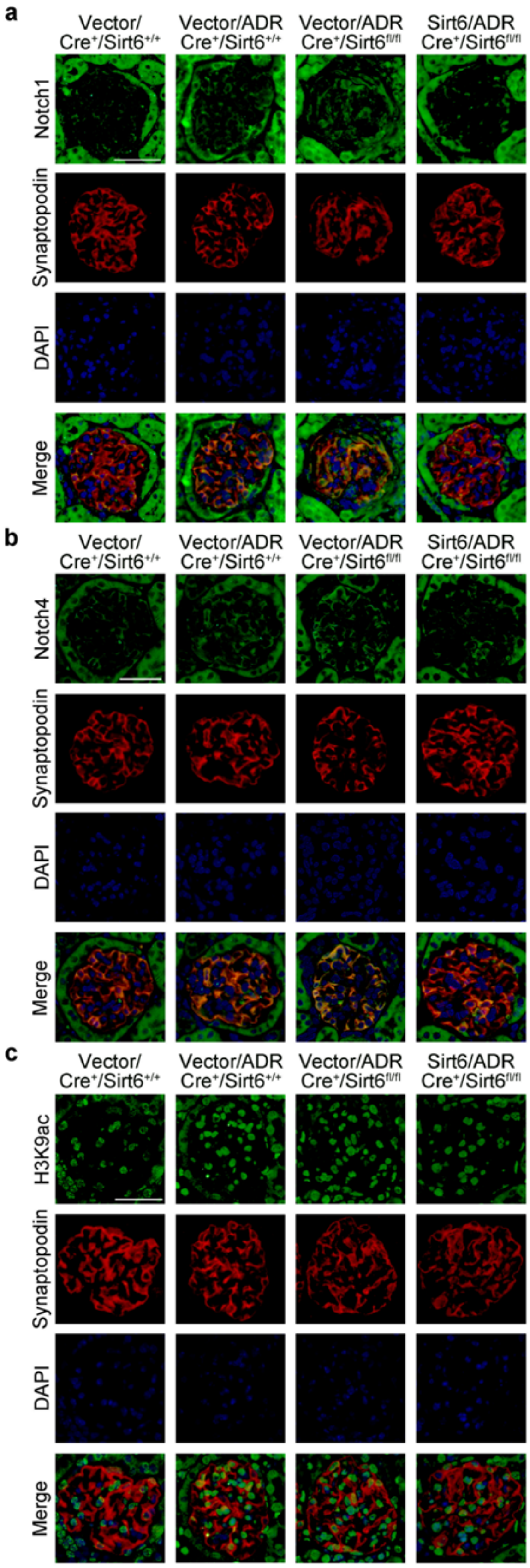

Supplementary Figure 11: Overexpression of Sirt6 reduced the levels of Notch1, Notch4 and H3K9ac in podocytes from mice with ADR treatment. **(a)** Representative confocal microscopic images showing the expression level of Notch1 in podocytes from different groups of mice. **(b)** Representative confocal microscopic images showing the expression level of Notch4 in podocytes from different groups of mice. **(c)** Representative confocal microscopic images showing the level of H3K9ac in podocytes from different groups of mice, synaptopodin was used as a podocyte marker. Scale bar, 25 µm.

Fig.1a

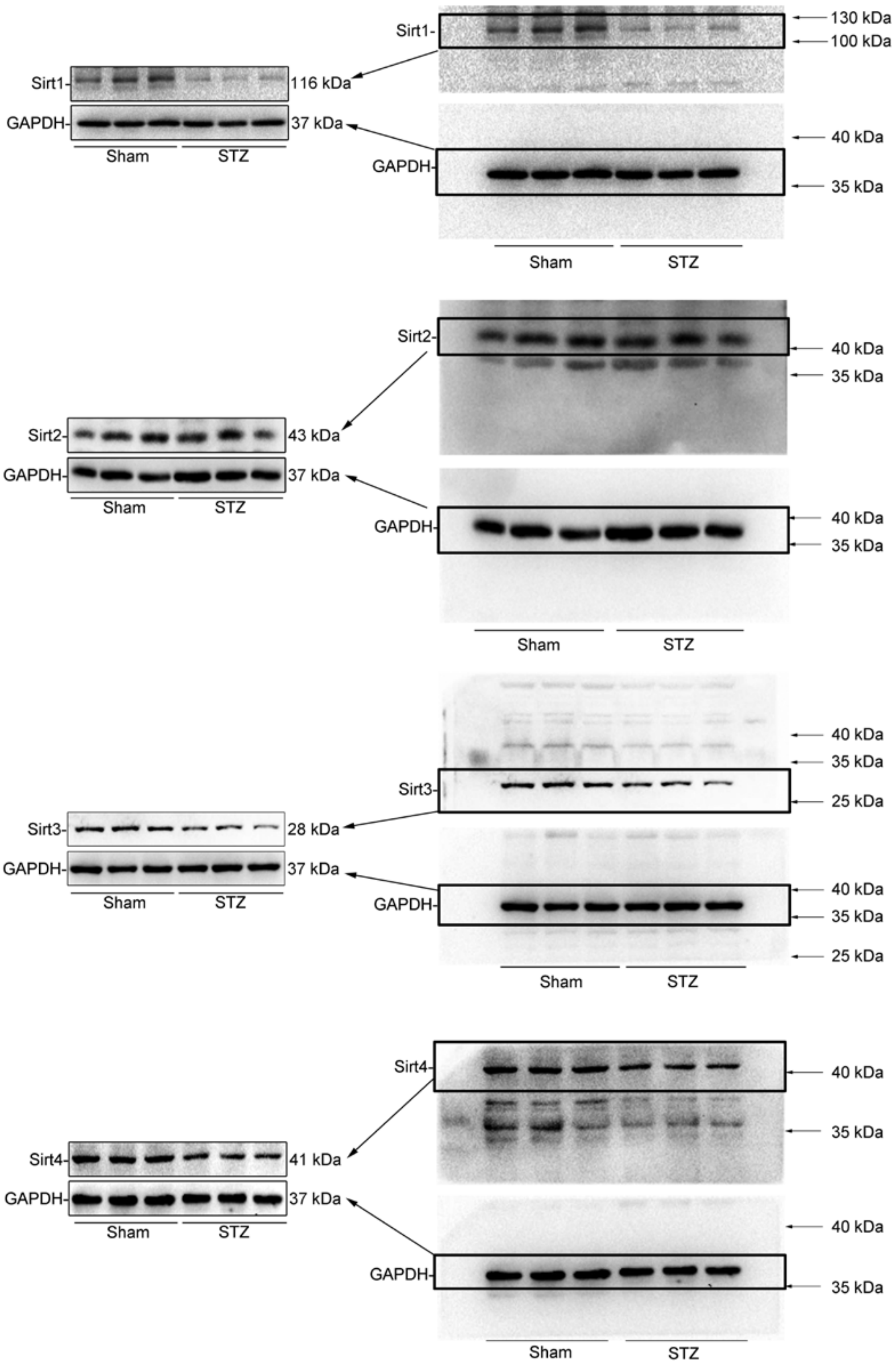
Sirt1-
116 kDa
GAPDH-
37 kDa
Sham
STZ
Sirt1-
130 kDa
100 kDa
GAPDH-
40 kDa
35 kDa
Sham
STZ
Sirt2-
43 kDa
GAPDH-
37 kDa
Sham
STZ
Sirt2-
40 kDa
35 kDa
GAPDH-
40 kDa
35 kDa
Sham
STZ
Sirt3-
28 kDa
GAPDH-
37 kDa
Sham
STZ
40 kDa
35 kDa
Sirt3-
25 kDa
GAPDH-
40 kDa
35 kDa
25 kDa
Sham
STZ
Sirt4-
41 kDa
GAPDH-
37 kDa
Sham
STZ
Sirt4-
40 kDa
35 kDa
GAPDH-
40 kDa
35 kDa
Sham
STZ

Fig.1a

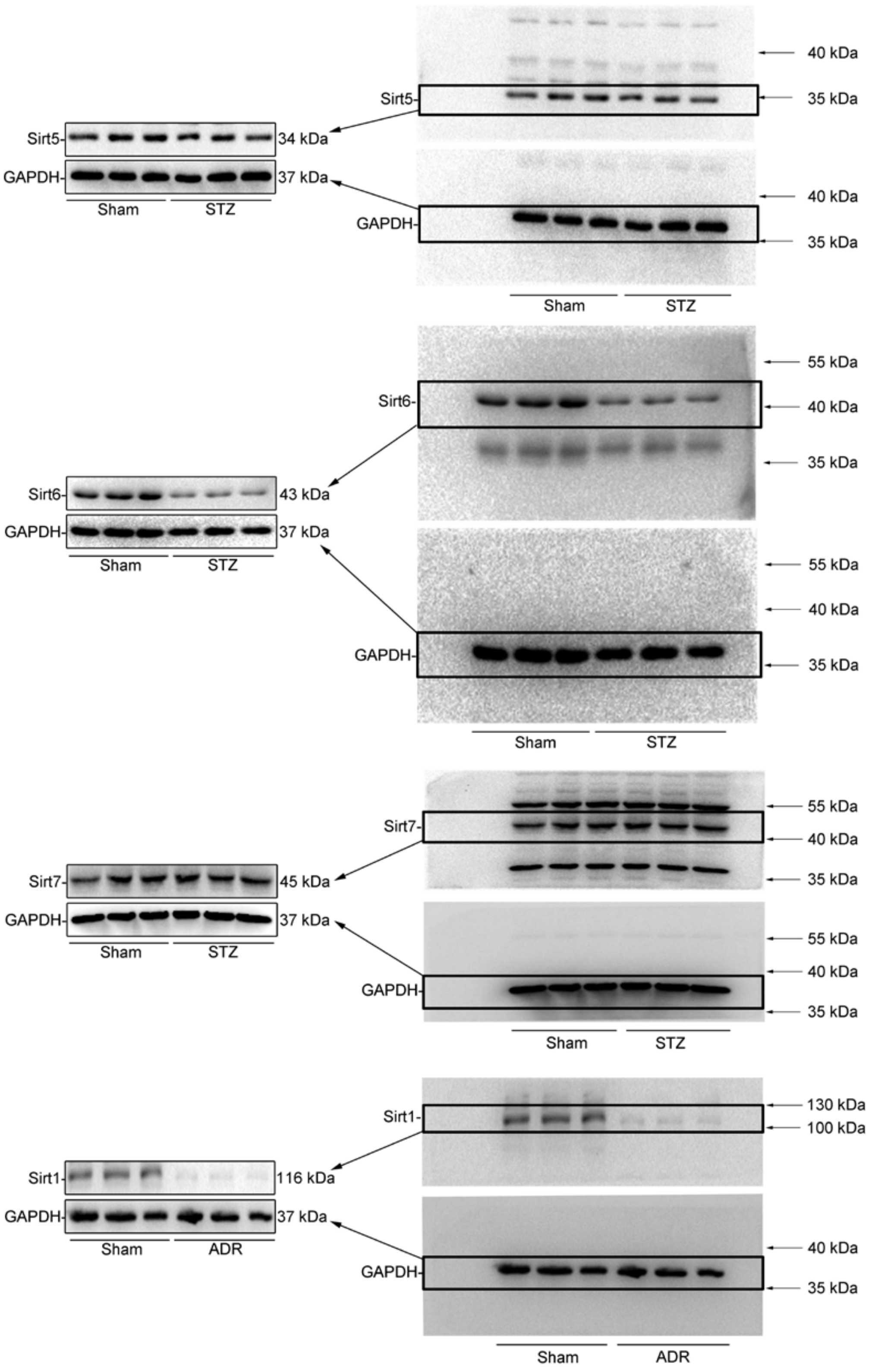
Sirt5
34 kDa
GAPDH
37 kDa
Sham
STZ
40 kDa
35 kDa
Sirt6
43 kDa
55 kDa
ADR
Sirt7
45 kDa
Sirt1
116 kDa
130 kDa
100 kDa

Fig.1a

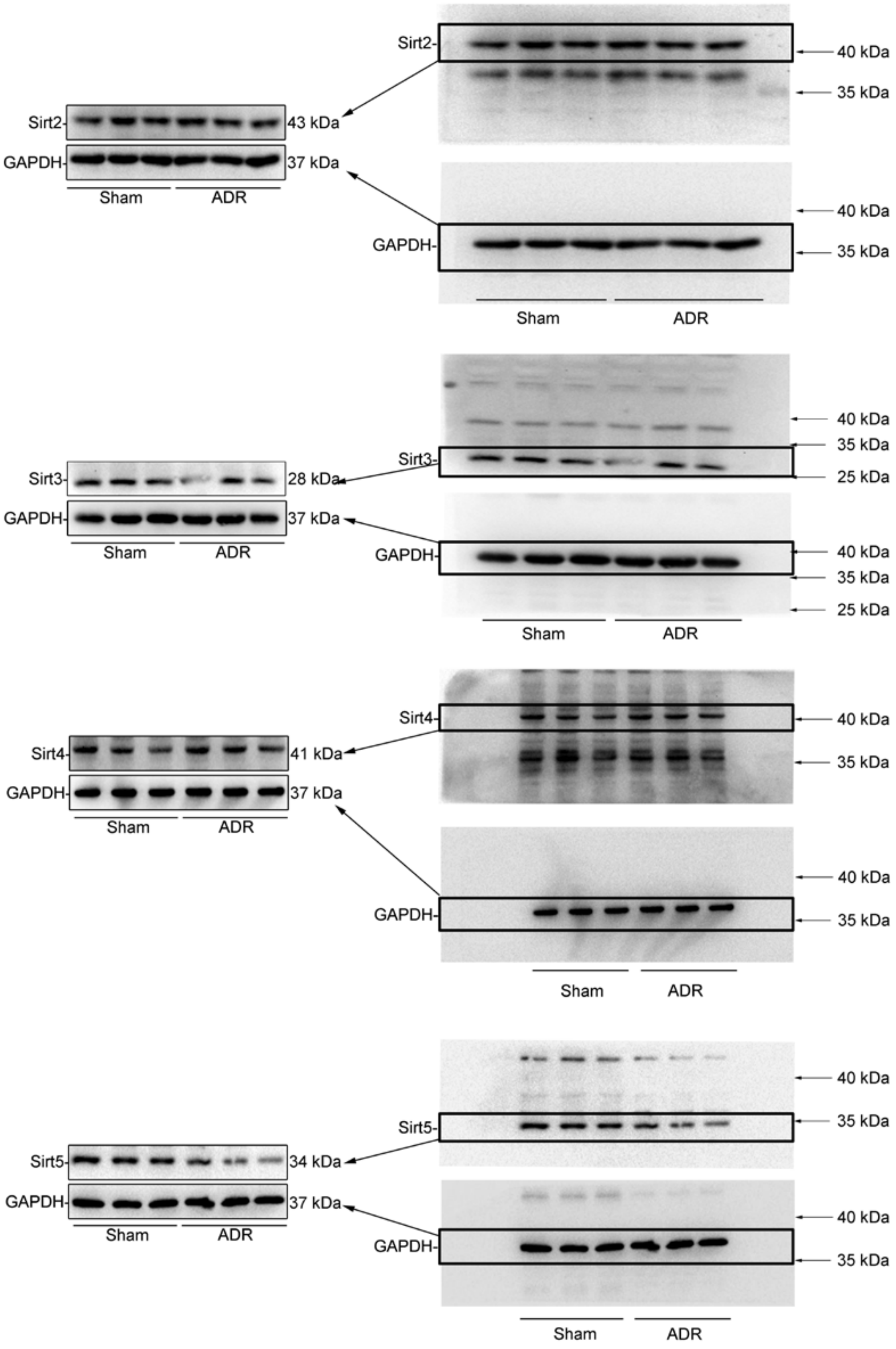
Sirt2
40 kDa
35 kDa
Sirt2
43 kDa
GAPDH
37 kDa
Sham
ADR
40 kDa
GAPDH
35 kDa
Sham
ADR
40 kDa
35 kDa
Sirt3
25 kDa
Sirt3
28 kDa
GAPDH
37 kDa
Sham
ADR
GAPDH
40 kDa
35 kDa
25 kDa
Sham
ADR
Sirt4
40 kDa
Sirt4
41 kDa
35 kDa
GAPDH
37 kDa
Sham
ADR
40 kDa
GAPDH
35 kDa
Sham
ADR
40 kDa
Sirt5
35 kDa
Sirt5
34 kDa
GAPDH
37 kDa
Sham
ADR
40 kDa
GAPDH
35 kDa
Sham
ADR

Fig.1a

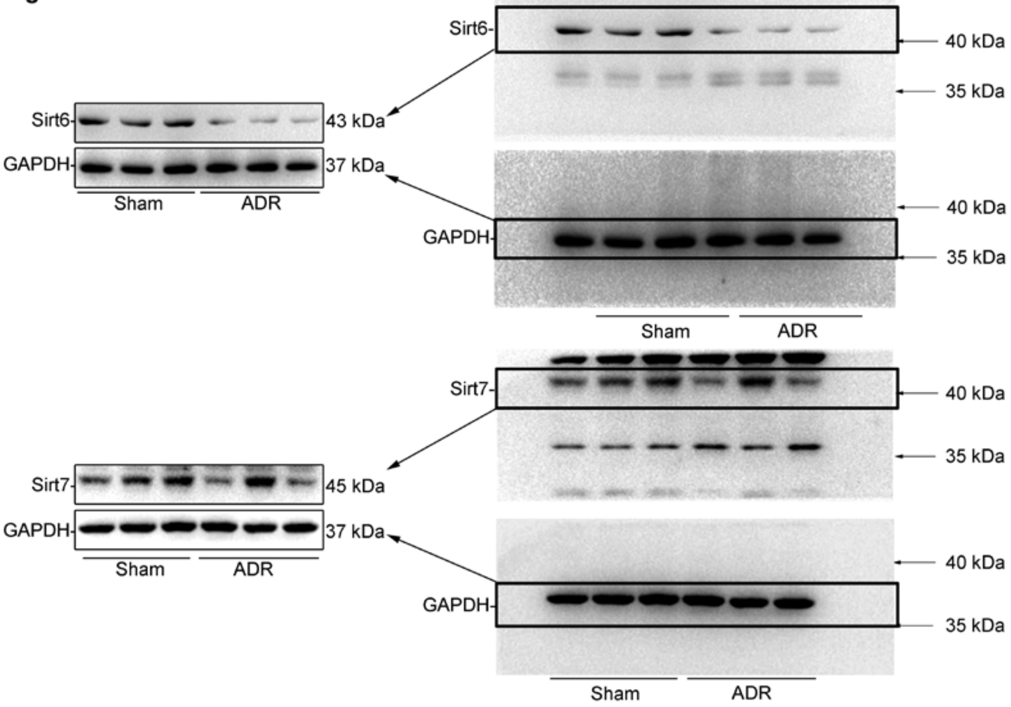

Fig.1b

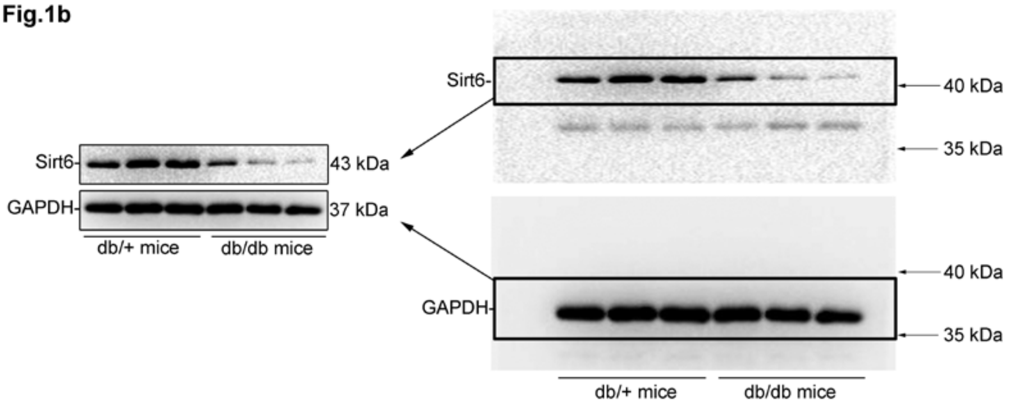

Fig.1d

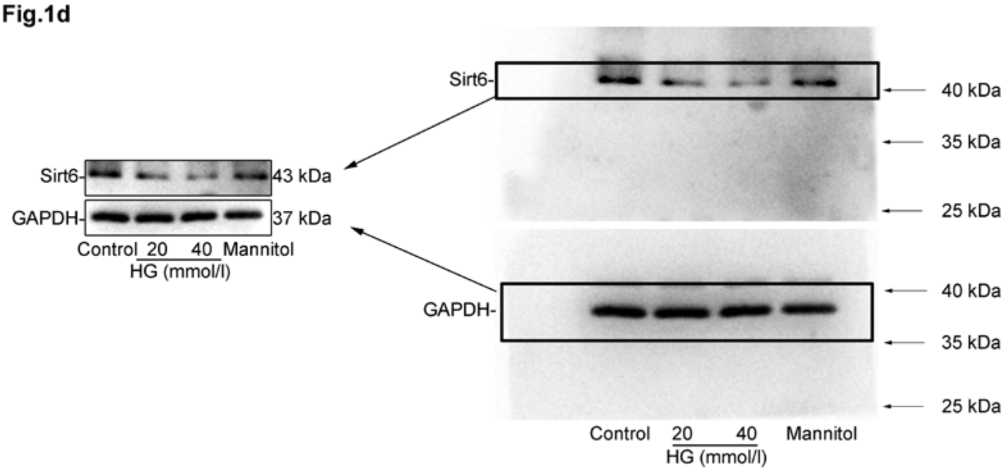

Fig.1e

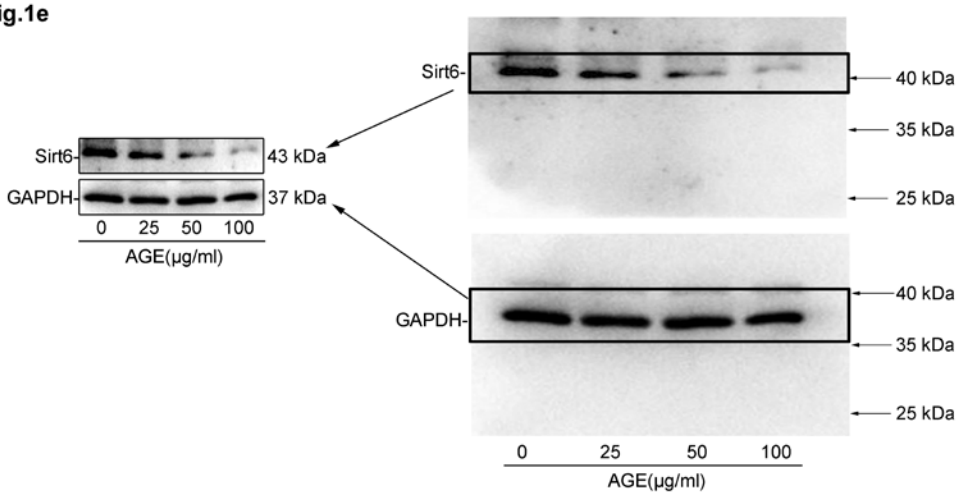

Fig.1f

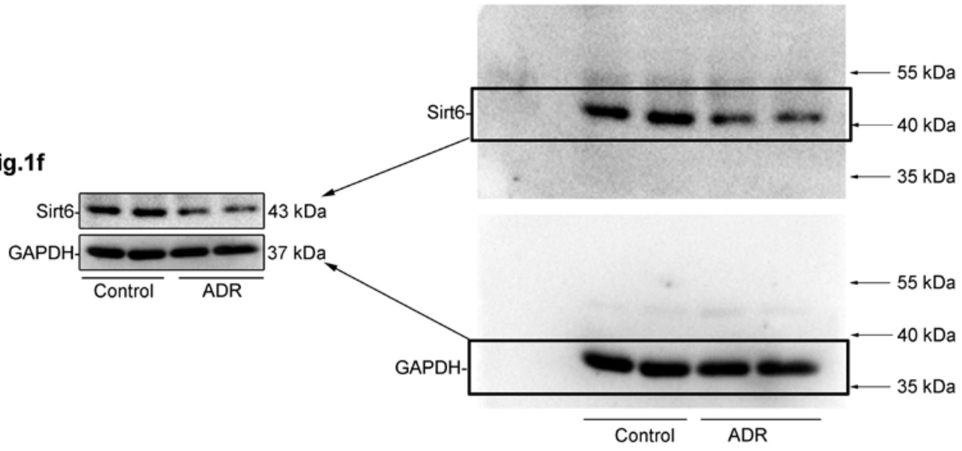

Fig.3a

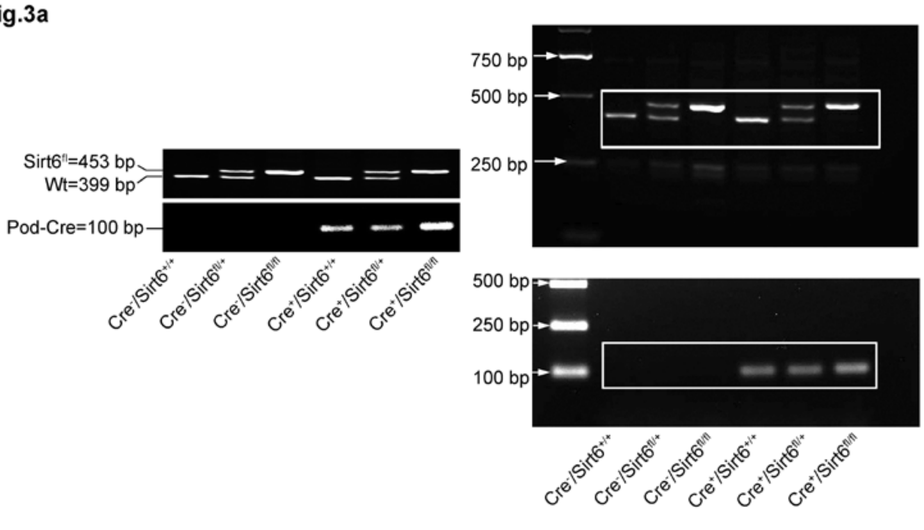

Fig.3c

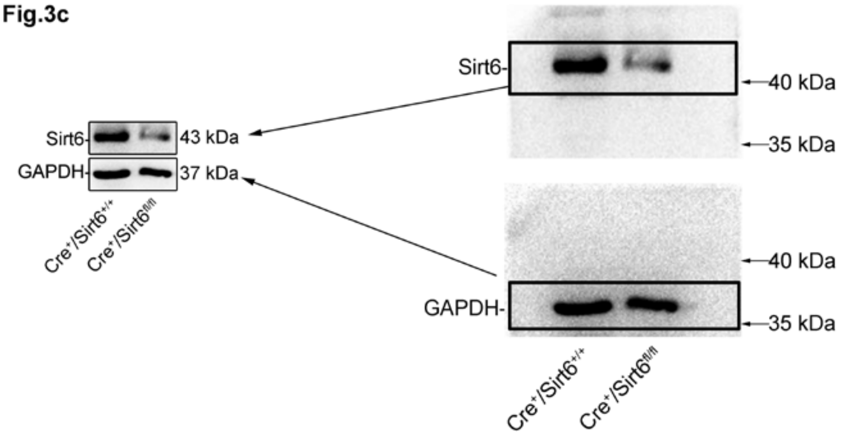

Fig.5a

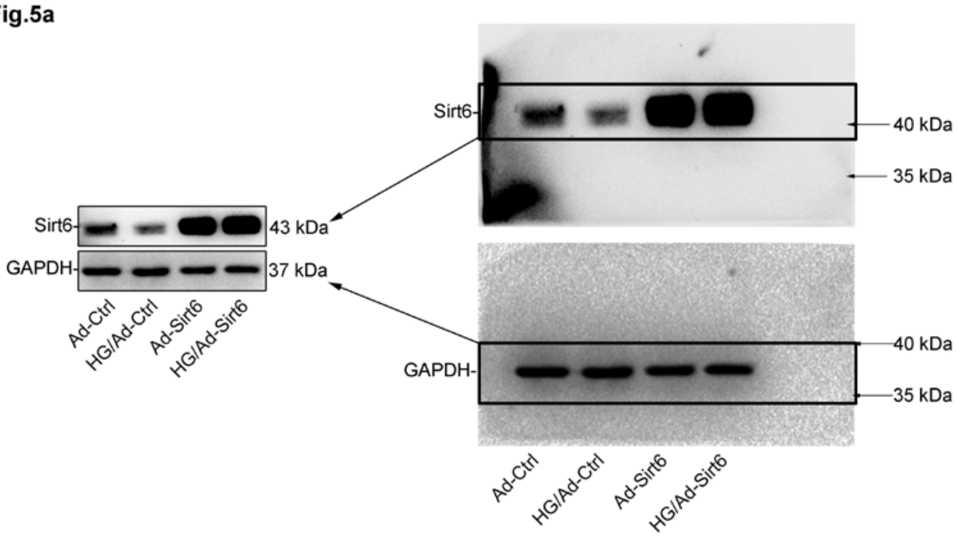

Fig.5e

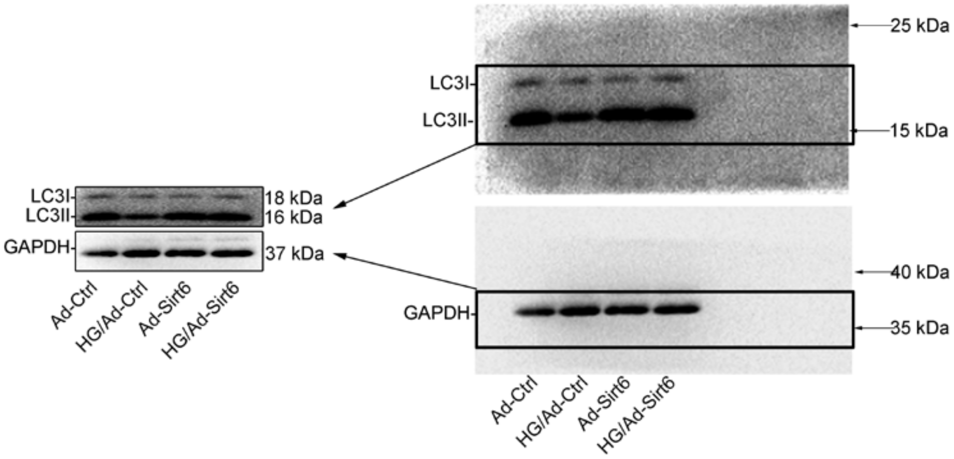

Fig.5e

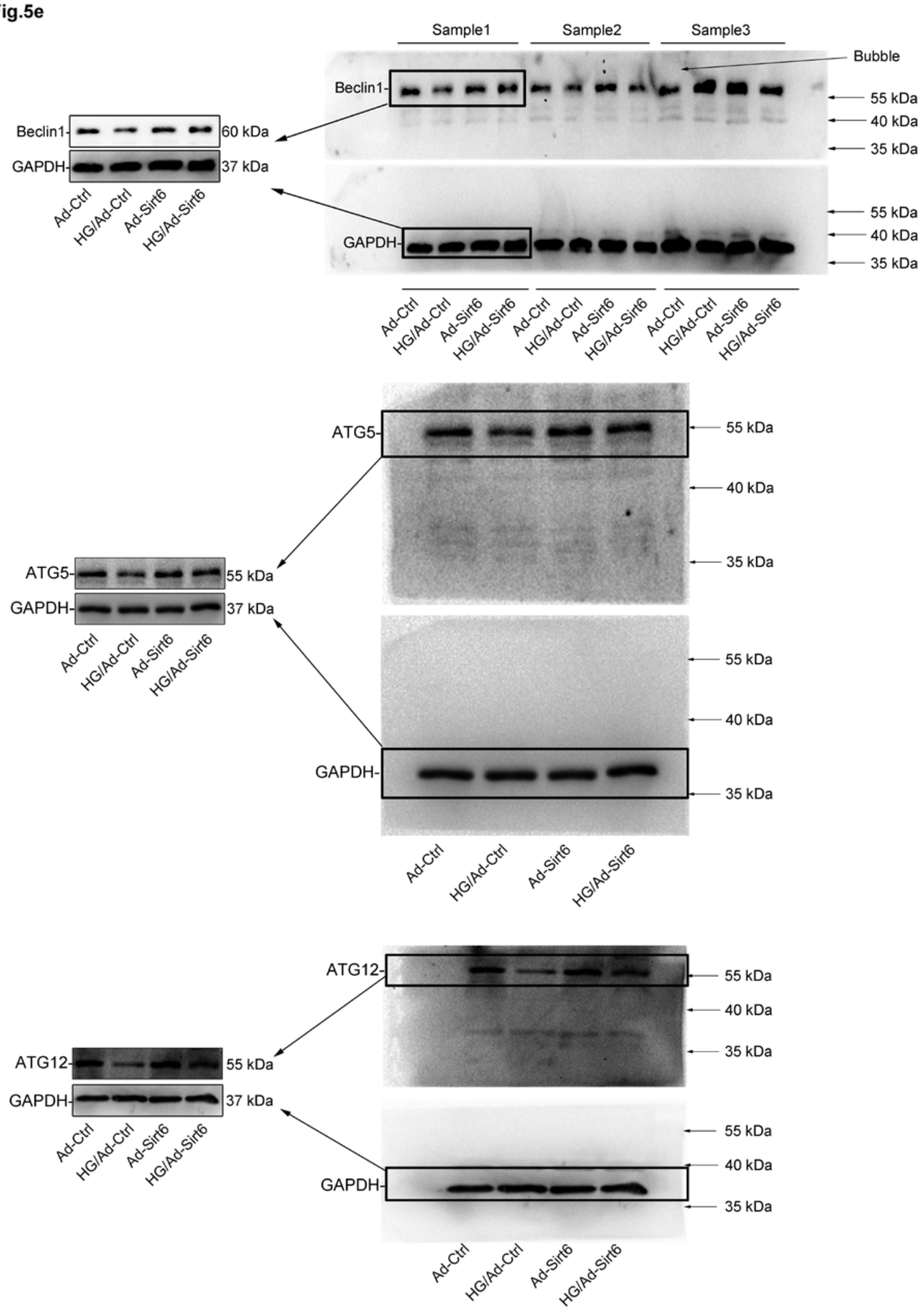
Sample1
Sample2
Sample3
Bubble
Beclin1-
55 kDa
40 kDa
35 kDa
Beclin1-
60 kDa
GAPDH-
37 kDa
Ad-Ctrl
HG/Ad-Ctrl
Ad-Sirt6
HG/Ad-Sirt6
GAPDH-
ATG5-
ATG5-
55 kDa
37 kDa
ATG12-
ATG12-

Fig.6c

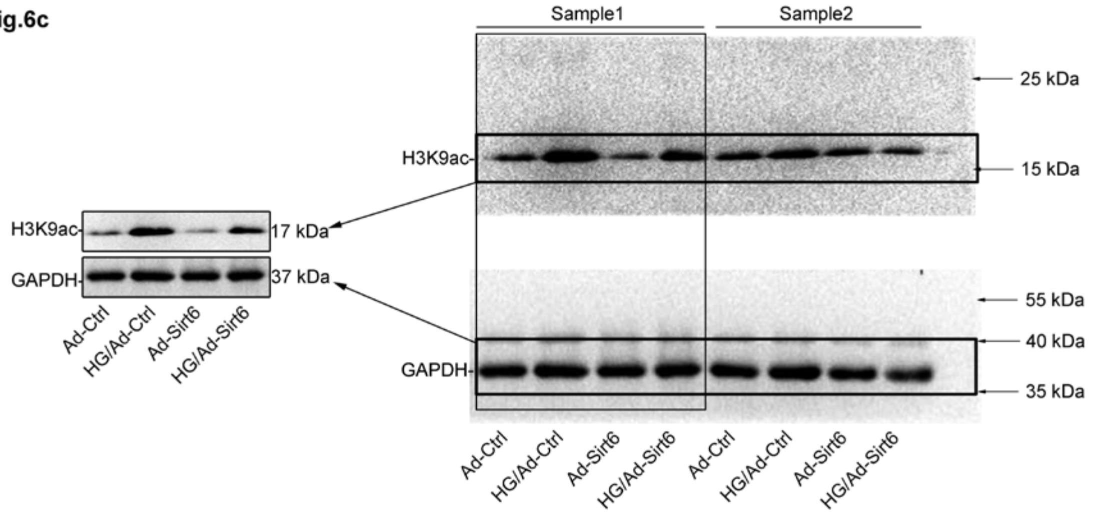
Sample1
Sample2
H3K9ac
GAPDH
17 kDa
37 kDa
25 kDa
15 kDa
55 kDa
40 kDa
35 kDa
Ad-Ctrl
HG/Ad-Ctrl
Ad-Sirt6
HG/Ad-Sirt6

Fig.6i

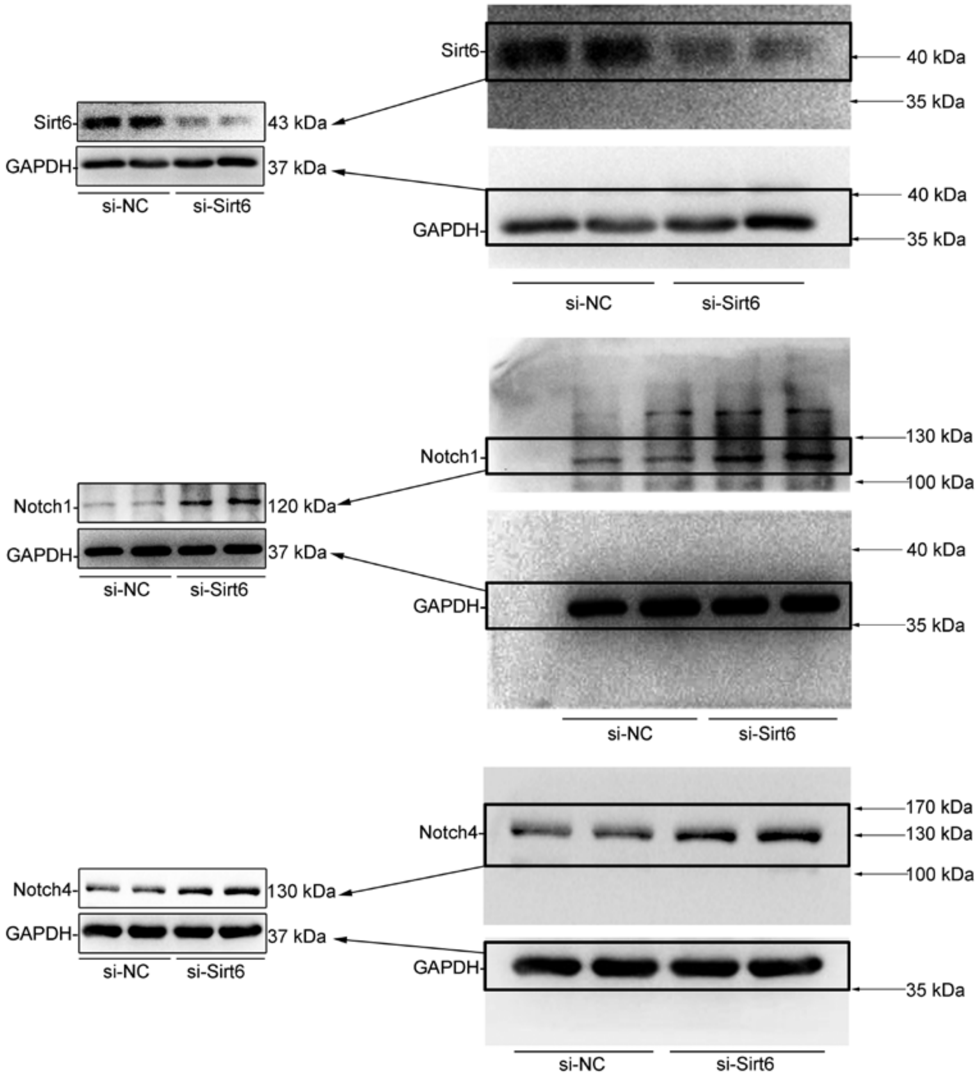
Sirt6
GAPDH
43 kDa
37 kDa
40 kDa
35 kDa
si-NC
si-Sirt6
Notch1
120 kDa
130 kDa
100 kDa
Notch4
170 kDa

Fig.7d

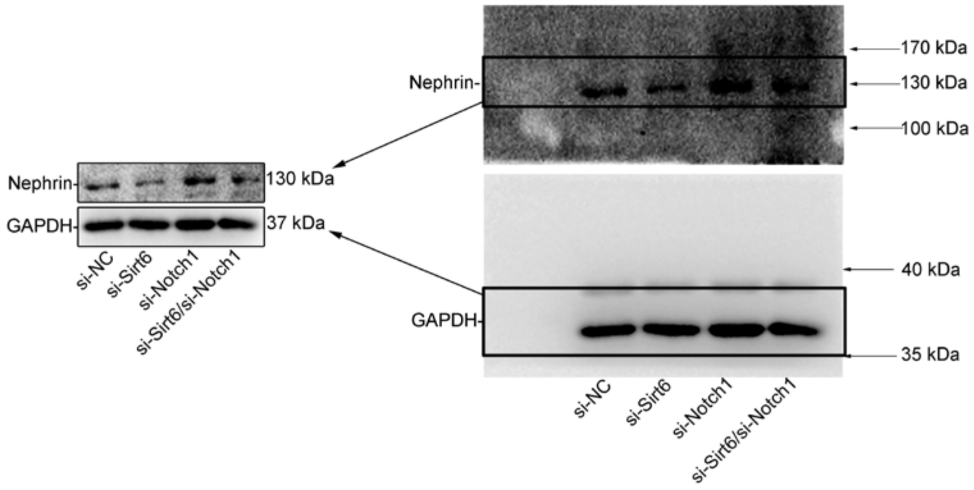

Nephrin
130 kDa
GAPDH
37 kDa
si-NC
si-Sirt6
si-Notch1
si-Sirt6/si-Notch1
170 kDa
130 kDa
100 kDa
40 kDa
35 kDa

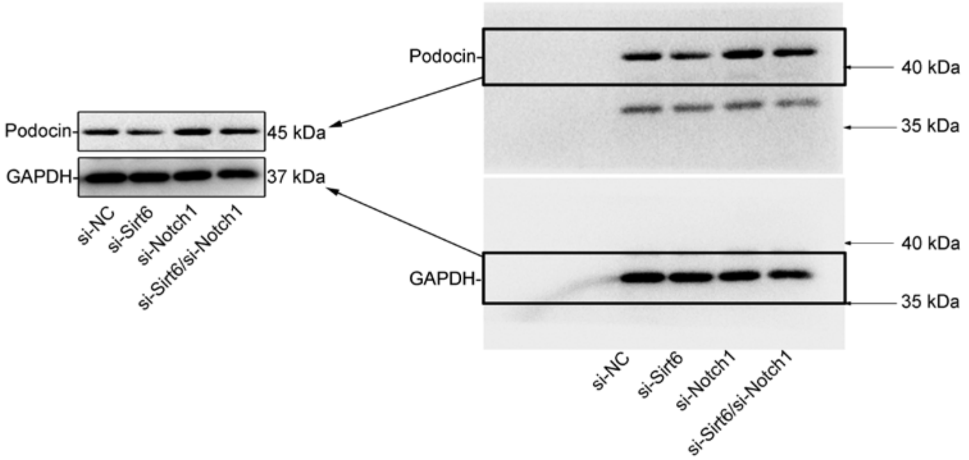

Podocin
45 kDa
GAPDH
37 kDa
si-NC
si-Sirt6
si-Notch1
si-Sirt6/si-Notch1
40 kDa
35 kDa
40 kDa
35 kDa

Fig.7e

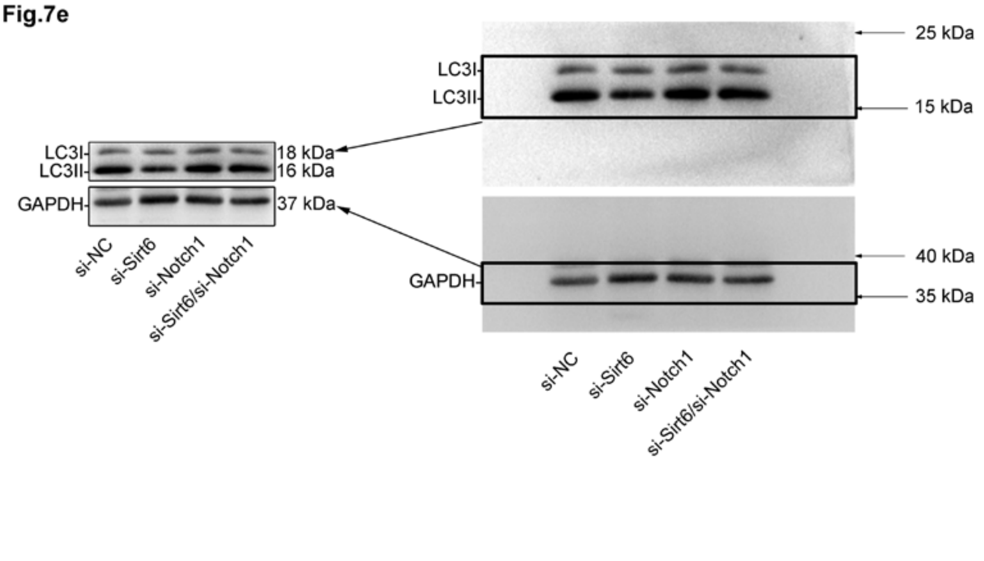

LC3I
LC3II
18 kDa
16 kDa
GAPDH
37 kDa
si-NC
si-Sirt6
si-Notch1
si-Sirt6/si-Notch1
25 kDa
15 kDa
40 kDa
35 kDa

Fig.7e

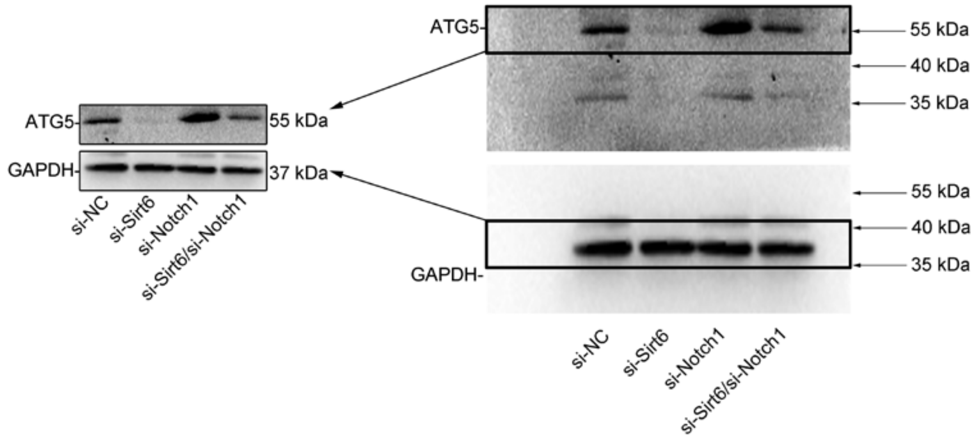

Supplementary Figure 12.
Scans of the full films used to generate Western blot data for figures 1-7

Supplementary figure 2

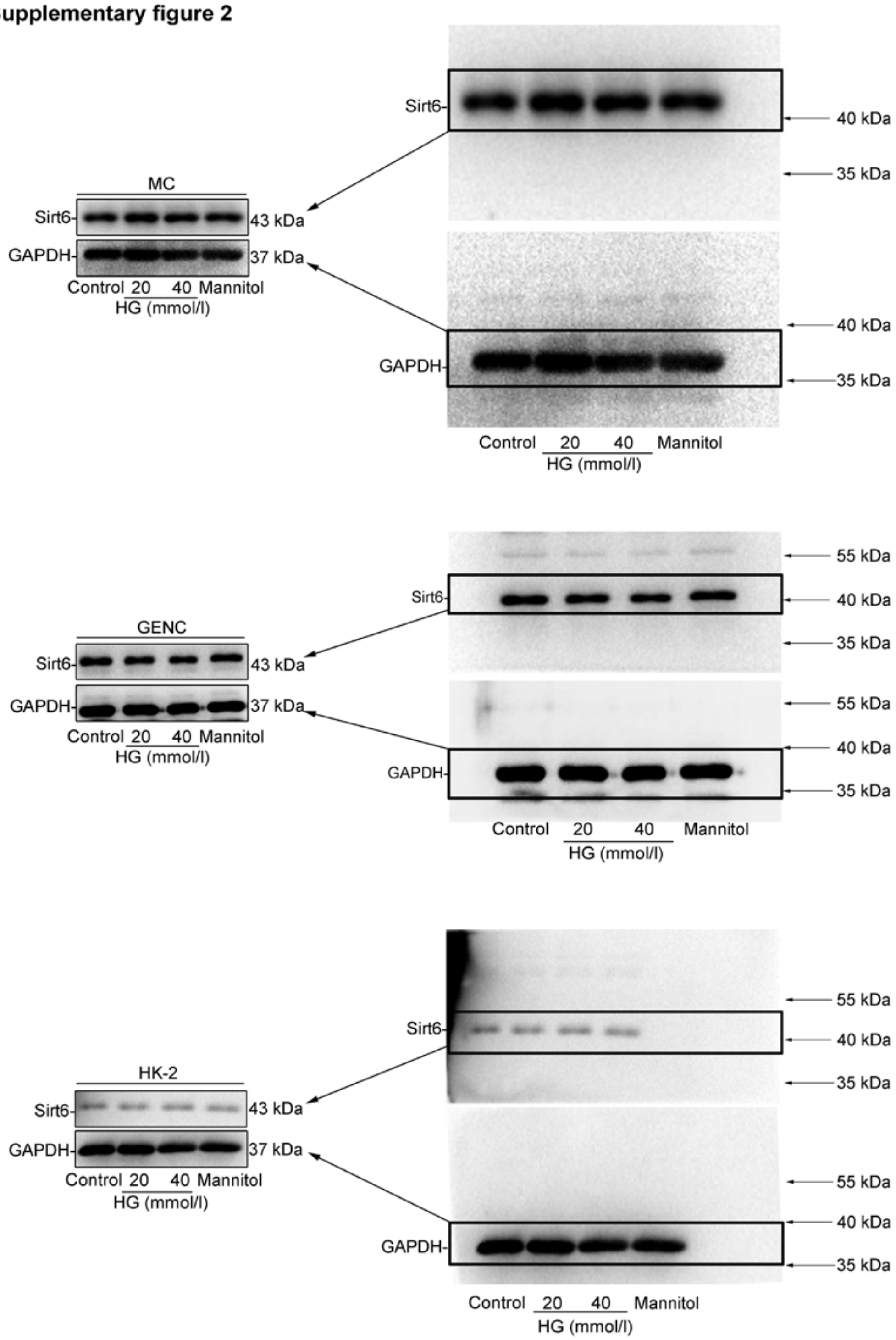

Supplementary figure 3

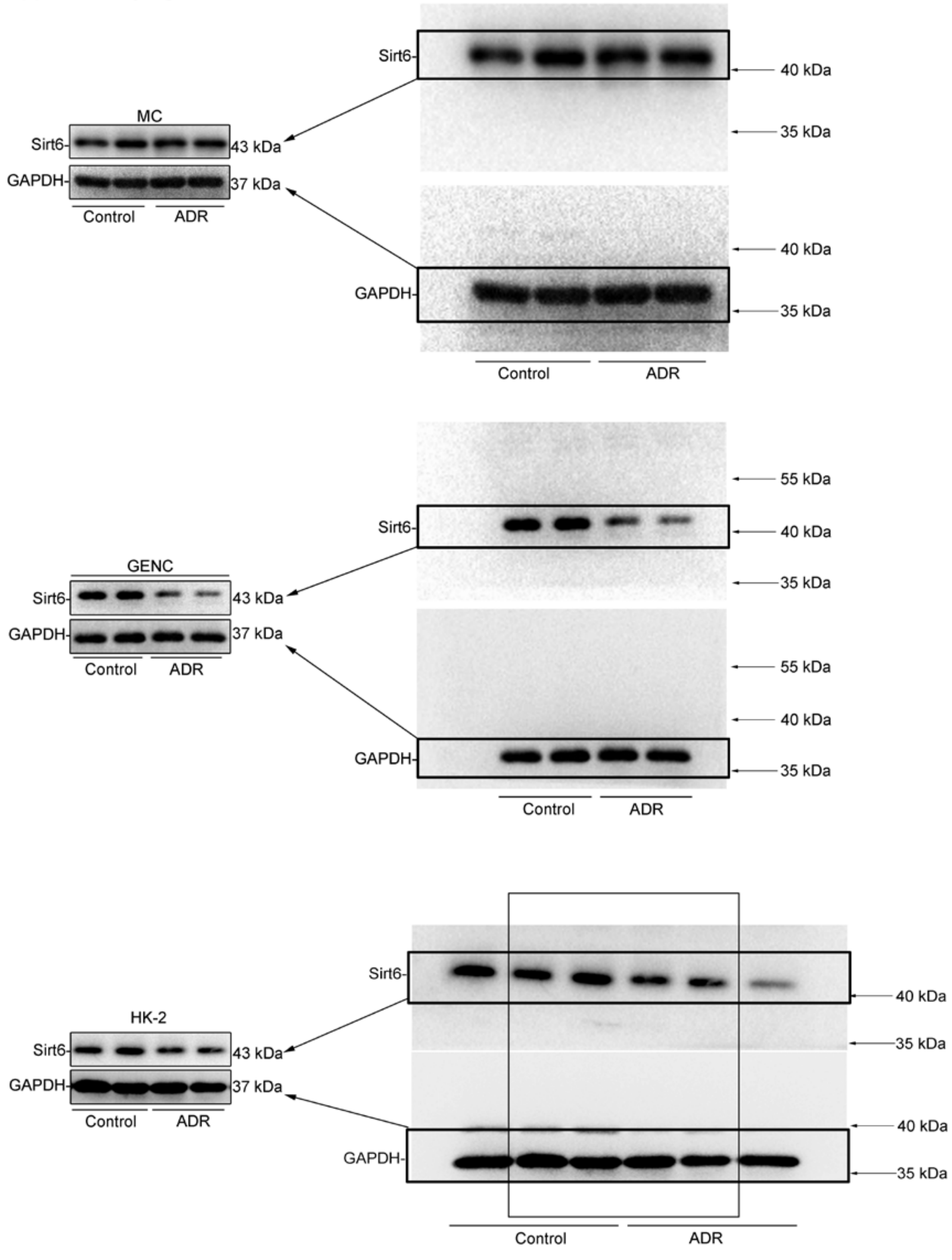

Supplementary figure 4

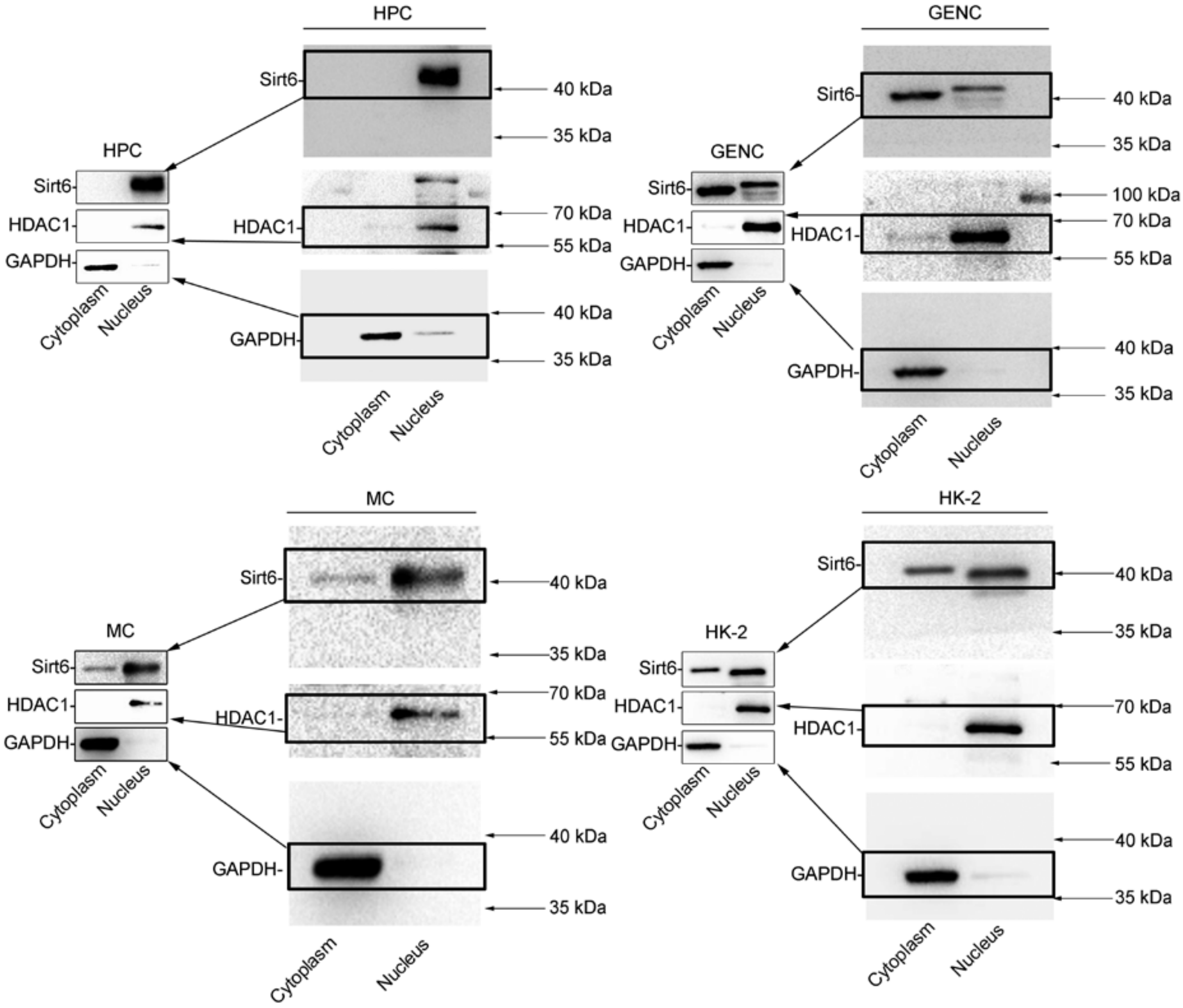

Supplementary figure 5a

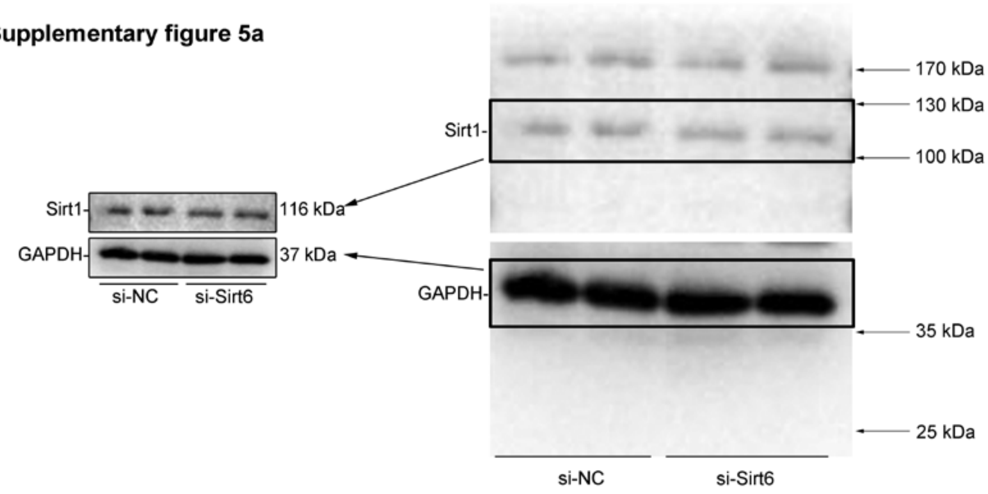

Supplementary figure 5a

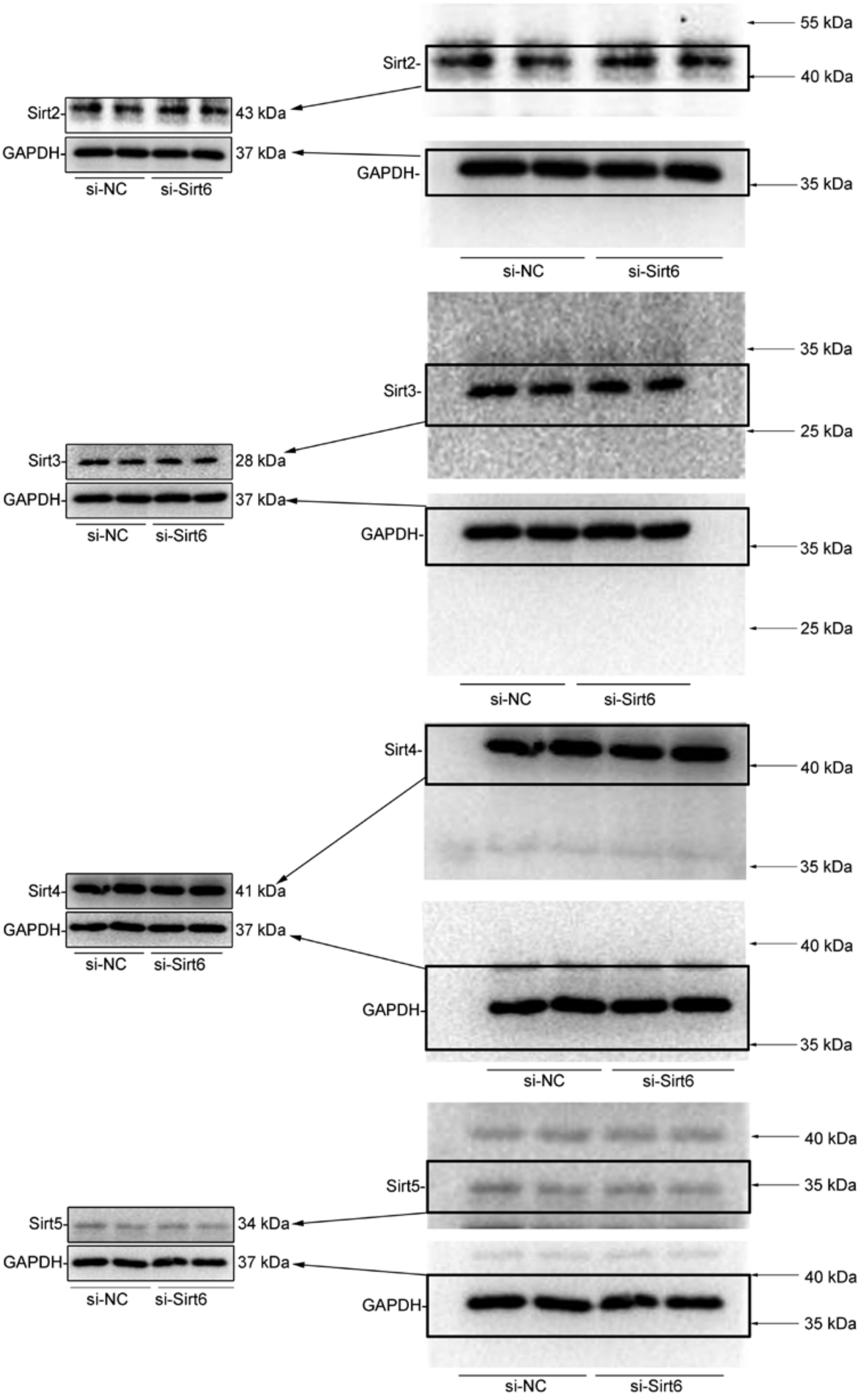

Supplementary figure 5a

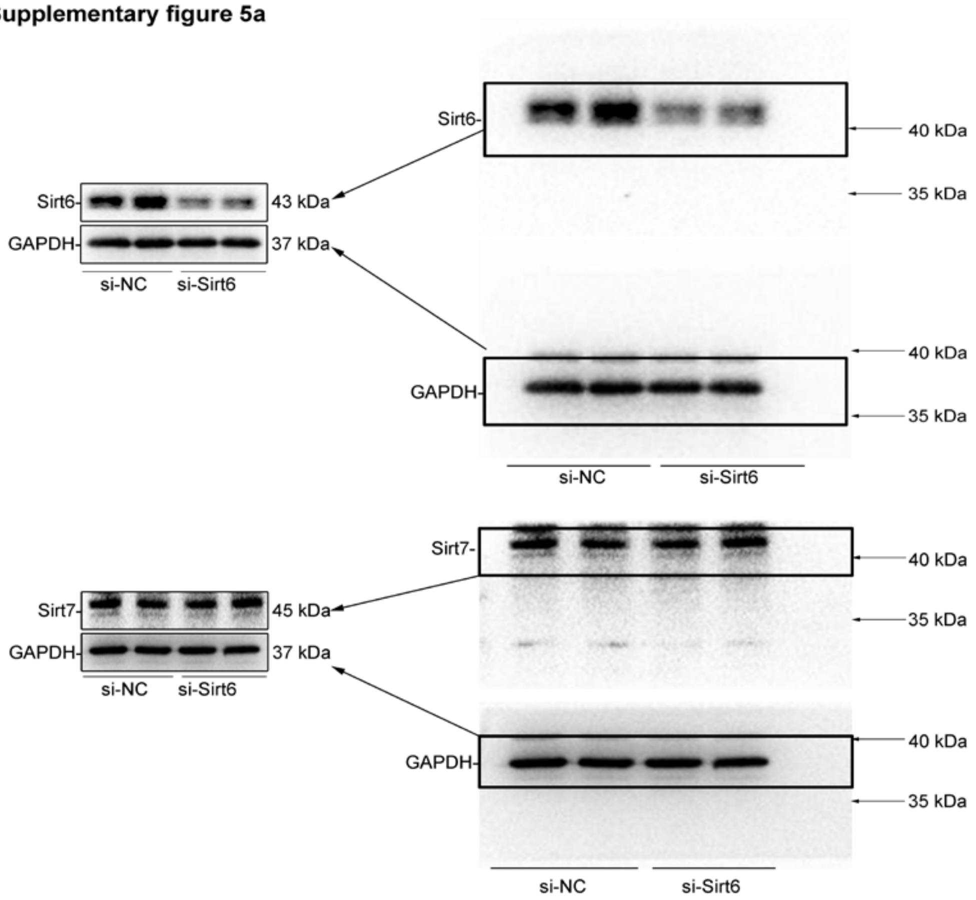

Supplementary figure 5b

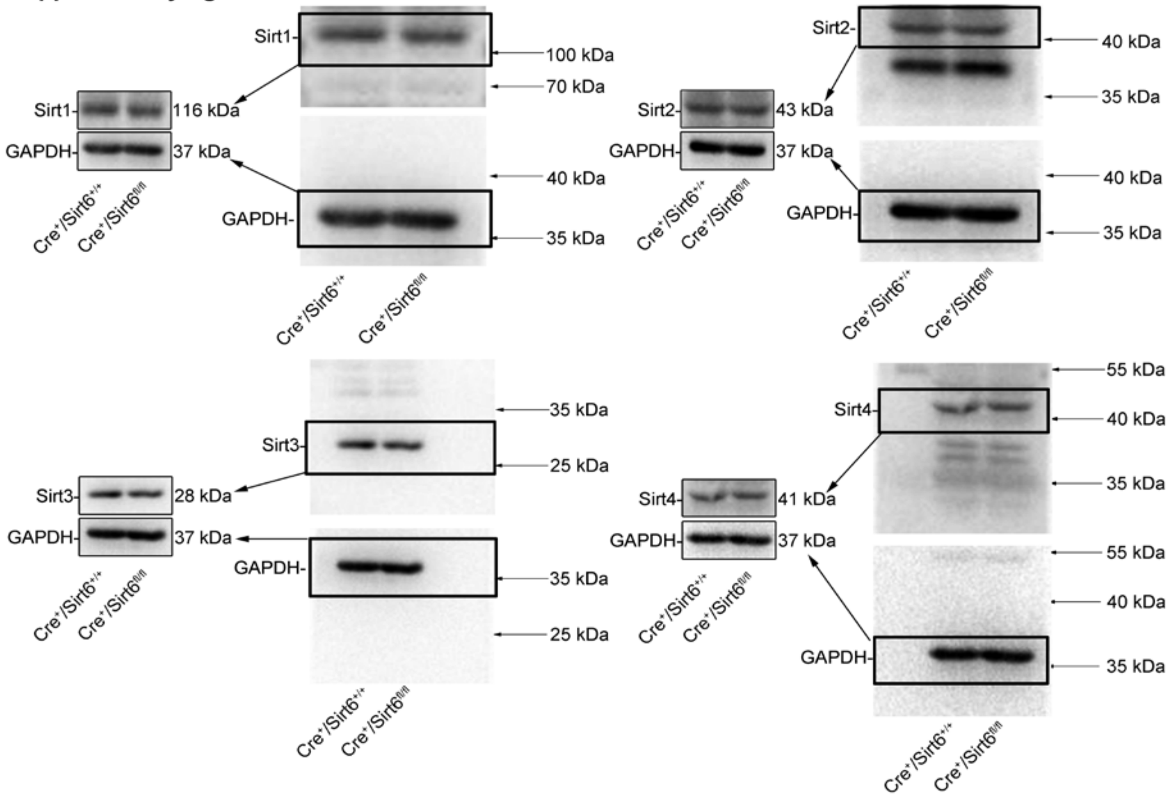

Supplementary figure 5b

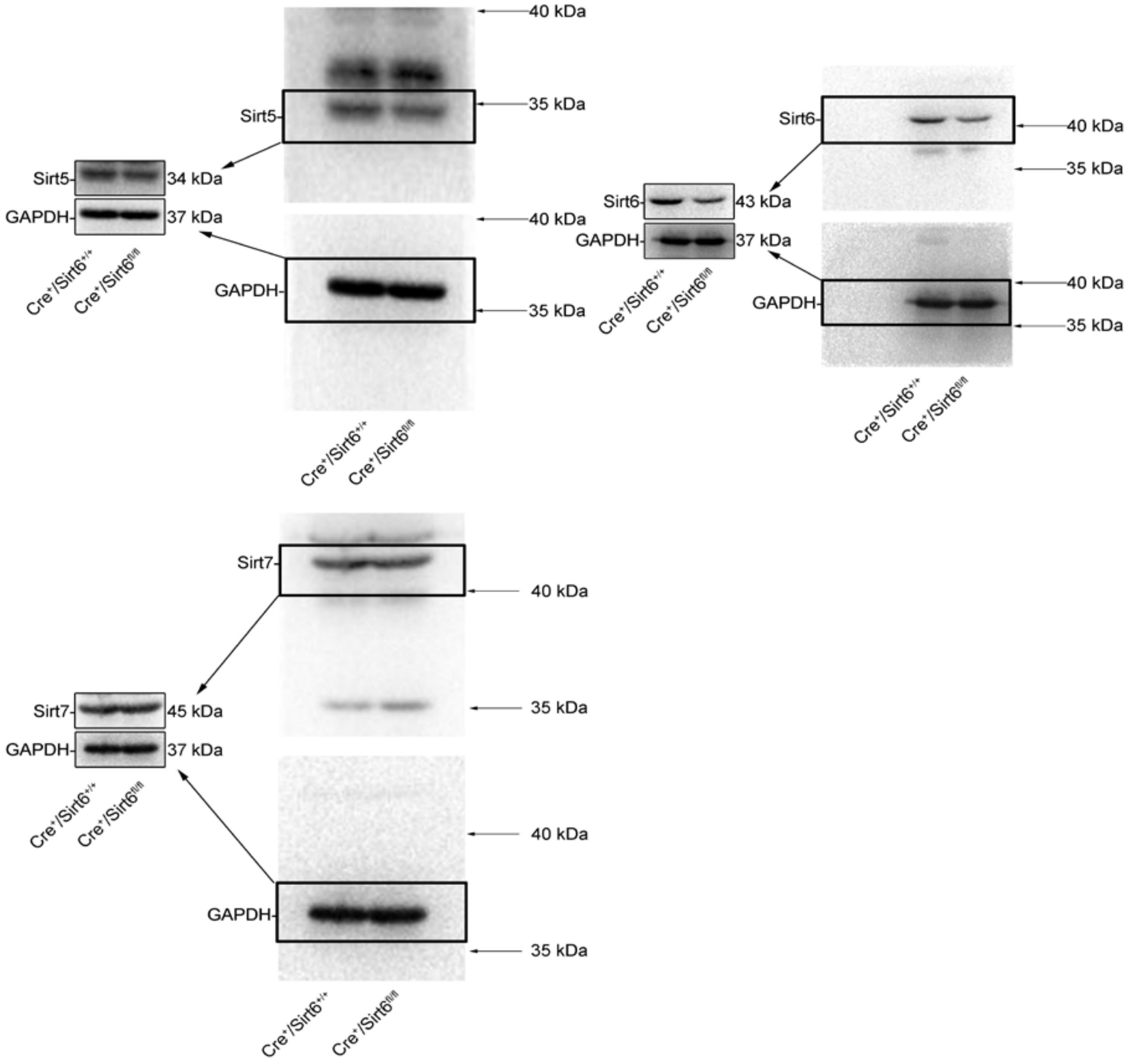

Supplementary figure 6a

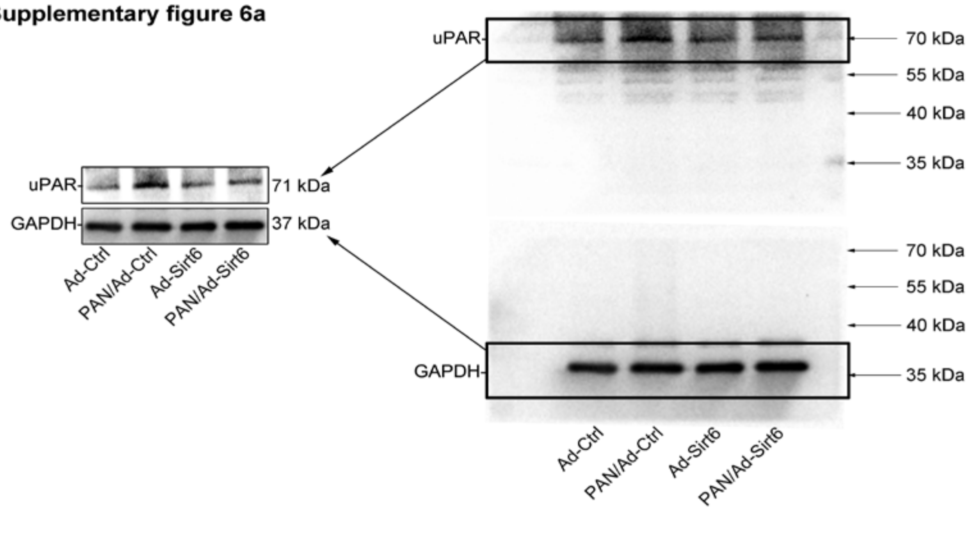

Supplementary figure 6b

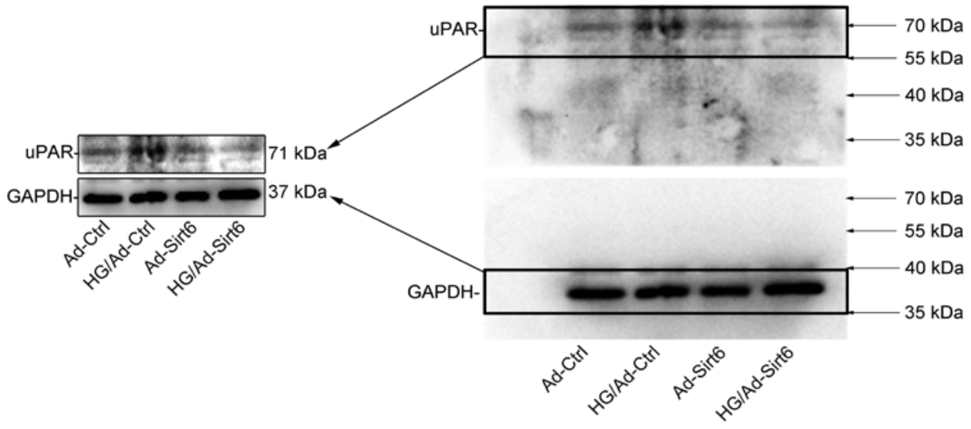

Supplementary figure 8

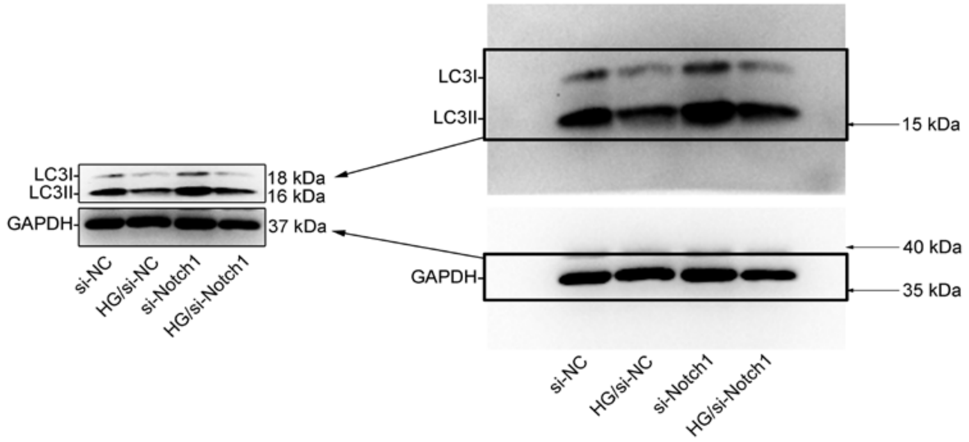

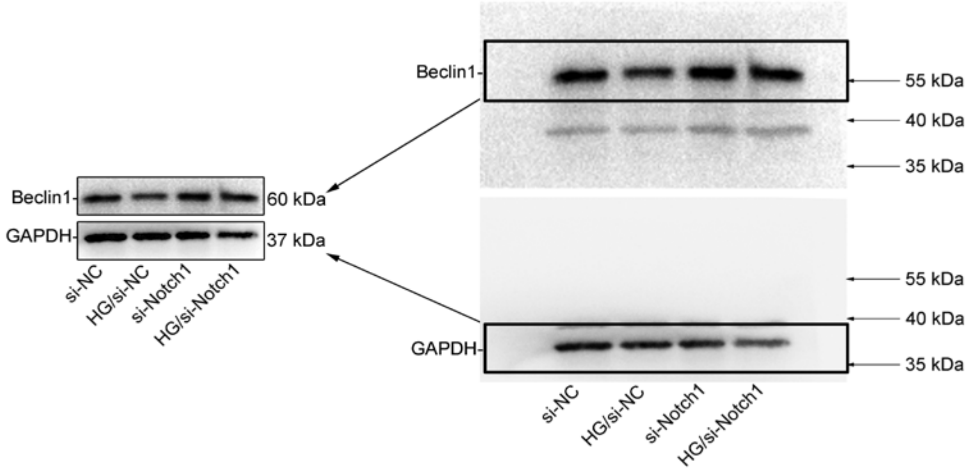

Supplementary figure 8

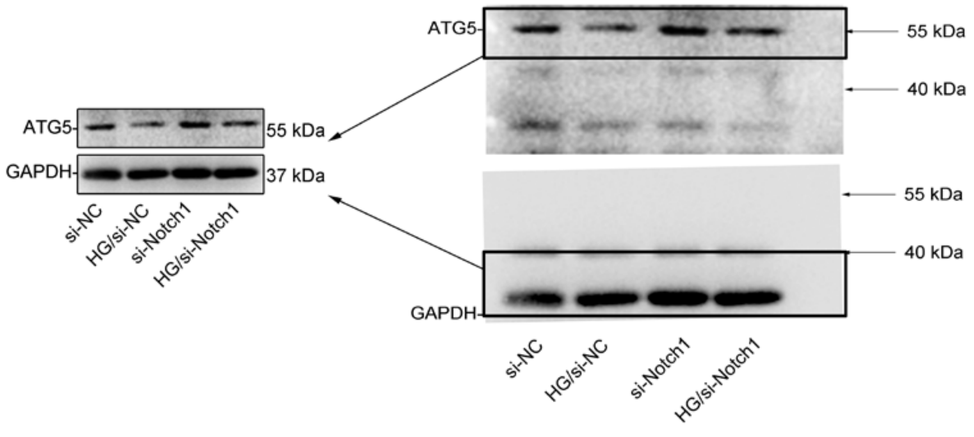

Supplementary Figure 13.
Scans of the full films used to generate Western blot data for Supplementary figures 1-12.

Supplementary Table 1. Physical and biochemical parameters of experimental animals.

Variable		*Cre⁺/SIRT6⁺/⁺*		*Cre⁺/SIRT6^fl/fl*	
		**Sham**	**STZ**	**Sham**	**STZ**
**Body weight (g)**		29.4 ± 4.1	22.8 ± 5.53*	28.5 ± 2.67	19.9 ± 5.36*
**kidney weight (g)**		0.31 ± 0.06	0.39 ± 0.07*	0.31 ± 0.03	0.40 ± 0.06*
**Heart rate (beat/min)**		478 ±7.61	469 ±8.79	473 ± 6.69	463 ± 11.8
**Blood pressure (mm Hg)**	**Systolic**	108.1 ± 8.9	110.3 ± 9.8	107.9 ± 11.8	112.8 ± 9.3
	**Diastolic**	68.7 ± 10.1	70.3 ± 8.1	68.3 ± 13.7	71.8 ± 9.1
**Glucose (mmol/l)**		6.0± 0.92	20.5 ± 2.46*	6.4 ± 0.98	21.1 ± 3.88*

Values are means ± S.E.M. for 8 mice in each group. *P<0.05 vs. control mice. One-way ANOVA followed by Tukey's post-test for multiple comparisons was used for groups of three or more.

Supplementary Table 2. Clinical data and relative mRNA levels of Sirt6 in the kidney from patients with different forms of podocytopathies

Patient	Age at Biopsy(yr)	Sex	Scr (μmol/L)	Serum Urea (mmol/L)	UPE/24h (g)	eGFR (ml/min/1.73 m2)	CRP (mg/dl)	HbA1c (%)	Relative SIRT6 Expression
**Normal controls**									
1	67	M	80	3.92	<0.15	89	NA	NA	1
2	70	F	51	4.69	<0.15	110	NA	NA	1.47
3	39	M	44	4.12	<0.15	198	NA	NA	2.29
4	76	F	83	7.21	<0.15	62	NA	NA	0.79
5	55	F	64	4.91	<0.15	89	NA	NA	0.89
6	48	M	61	7.51	<0.15	130	NA	NA	2.43
7	51	M	46	3.51	<0.15	178	NA	NA	2.27
8	67	F	87	5.91	<0.15	60	NA	NA	0.78
9	49	M	59	4.12	<0.15	135	NA	NA	1.56
mean±SD	58.0±12.4		63.9±16.1	5.1±1.45		116.8±48.3			1.5±0.68
**FSGS**									
1	47	M	168	11.70	6.78	41	NA	NA	0.27
2	56	F	103	7.16	3.89	51	NA	NA	0.43
3	51	M	216	17.80	5.21	30	NA	NA	1.32
4	27	M	112	22.15	5.22	73	NA	NA	0.25
5	42	F	132	82.10	7.76	41	NA	NA	0.38
6	23	F	78	14.20	2.78	84	NA	NA	1.22
7	37	M	92	13.50	4.12	85	NA	NA	0.61
8	62	F	207	8.59	3.17	22	NA	NA	0.46
9	29	M	76	9.22	2.16	112	NA	NA	0.59
10	41	M	105	7.15	3.2	72	NA	NA	0.41
mean±SD	41.5±12.8		128.9±51.1	19.4±22.6	4.9±2.9	61.1±28.6			0.59±0.38
**MGN**									
1	67	F	77	6.76	2.5	93	NA	NA	1.29
2	41	F	88	4.57	3.8	65	NA	NA	0.39
3	53	F	52	3.71	4.3	153	NA	NA	0.53
4	37	M	77	6.70	4.9	105	NA	NA	0.62
5	72	M	101	8.22	7.8	67	NA	NA	0.29
6	45	M	69	3.67	2.3	114	NA	NA	1.51
7	35	F	58	5.30	1.8	109	NA	NA	0.79
8	47	M	66	6.02	8.6	119	NA	NA	0.26
mean±SD	50.0±13.6		74.6±15.9	5.6±1.6	3.9±2.5	100.9±28.7			0.71±0.46

**IgA**									
1	35	F	89	4.90	3.73	67	NA	NA	0.49
2	27	M	132	6.80	6.78	60	NA	NA	0.57
3	40	M	116	9.20	5.8	64	NA	NA	0.38
4	31	F	92	3.19	3.7	66	NA	NA	0.72
5	44	M	139	5.20	5.8	51	NA	NA	0.52
6	21	F	77	4.30	1.17	118	NA	NA	0.88
7	29	M	169	6.20	4.89	44	NA	NA	1.23
8	58	M	127	8.90	7.7	54	NA	NA	0.31
9	71	F	97	5.70	4.9	52	NA	NA	0.41
mean±SD	39.6±16.0		115.3±29.3	6.0±2.0	4.9±1.9	64.0±21.7			0.61±0.29
**DN group**									
1	56	F	158	18.80	3.65	31	<3.19	11.6	0.51
2	67	F	138	8.61	6.42	35	NA	8.2	0.29
3	51	M	89	21.8	4.08	83	NA	7.1	0.45
4	48	M	165	18.7	7.29	41	NA	10.2	0.36
5	72	F	98	9.42	4.08	51	NA	8.2	0.49
6	65	M	212	4.40	4.41	29	NA	11.3	1.23
7	45	F	77	27.6	1.26	75	NA	6.5	1.41
8	77	M	81	6.00	3.4	63	NA	7.1	0.49
mean±SD	60.1±11.8		127.3±48.8	14.4±8.4	4.3±1.9	51.0±20.6		8.8±2.0	0.65±0.42
**DM-NN group**									
1	49	M	55	6.20	<0.15	146	NA	7.6	1.53
2	52	F	68	5.17	<0.15	84	NA	8.0	0.48
3	61	M	92	4.82	<0.15	77	NA	6.9	1.21
4	72	M	101	7.73	<0.15	67	NA	7.2	0.77
5	38	M	67	3.96	<0.15	122	NA	10.0	1.53
6	50	M	88	6.10	<0.15	85	NA	9.2	2.67
7	44	F	61	5.20	<0.15	98	NA	8.3	1.13
mean±SD	52.3±11.2		76.0±17.5	5.1±0.8		97.0±27.8		8.2±1.1	1.3±0.8

SCr, serum creatinine; UPE, urinary protein excretion; eGFR, estimated GFR; HbA1c, hemoglobin A1c; F, female; M, male; DPN, diabetic peripheral neuropathy; DR, diabetic retinopathy; IHD, ischemic heart disease; CVA, cerebrovascular accident; N, not present; NA, not applicable/not available.

Supplementary Table 3. Primers for chromatin immunoprecipitation assay

Gene	Species	Primer sequences
Notch1-Promoter	Human	Forward: CCTTCTGCCATCGCACTCAC<br>Reverse: CCTGTGCCAAGCCTGGTTAA
Notch2-Promoter	Human	Forward: GAGGAATTTATTGCGGTGTCGG<br>Reverse: TGTTCCTTTGGCTTGGAATGAC
Notch3-Promoter	Human	Forward: TCACAGAGGAAGTGGGTTGC<br>Reverse: CAGCCTCAGACCTCAGACAC
Notch4-Promoter	Human	Forward: CCCCAAAGTTGTCCTGGGTT<br>Reverse: TCCTTGGGATGCAGGGAATG
Notch1-Promoter	Mouse	Forward: ATGCCACCTCCTGACTTTCT<br>Reverse: TCAACTGGACCAACCAATCC
Notch4-Promoter	Mouse	Forward: CTGGGCTACAGAATGAGACC<br>Reverse: AAAAGACAACCATAAAGGGA

Supplementary Table 4. Primer pairs of target genes used for real time RT-PCR in this study

Gene	Species	Primer sequences
Sirt6-Loxp	Mouse	Forward: AGTGAGGGGCTAATGGGAAC<br>Reverse: AACCCACCTCTCTCCCCTAA
Podocin-Cre	Mouse	Forward: GCGGTCTGGCAGTAAAAACTATC<br>Reverse: GTGAAACAGCATTGCTGTCACTT
Sirt6	Human	Forward: AATTACGCGGCGGGGCT<br>Reverse: CGCGCGCTCTCAAAGGT
Sirt6	Mouse	Forward: GGCTACGTGGATGAGGTGAT<br>Reverse: GGCTCAGCCTTGAGTGCTAC
Nothc1	Human	Forward: GAGGCGTGGCAGACTATGC<br>Reverse: CTTGTACTCCGTCAGCGTGA
Notch2	Human	Forward: GGCATTAATCGCTACAGTTGTGTCT<br>Reverse: GGAGGCACACTCATCAATGTCA
Notch3	Human	Forward: CGTGGCTTCTTTCTACTGTGC<br>Reverse: CGTTCACCGGATTTGTGTCAC
Notch4	Human	Forward: TGTGAACGTGATGTCAACGAG<br>Reverse: ACAGTCTGGGCCTATGAAACC
IL-1β	Human	Forward: ATGATGGCTTATTACAGTGGCAA<br>Reverse: GTCGGAGATTCGTAGCTGGA
IL-6	Human	Forward: ACTCACCTCTTCAGAACGAATTG<br>Reverse: CCATCTTTGGAAGGTTCAGGTTG
TNF-α	Human	Forward: CCTCTCTCTAATCAGCCCTCTG<br>Reverse: GAGGACCTGGGAGTAGATGAG
β-actin	Human	Forward: GAAGTGTGACGTGGACATCC<br>Reverse: CCGATCCACACGGAGTACTT

Supplementary Table 5. Antibodies used in this study

Primary antibodies	Host	Dilution and supplier	Product ID	Application
Sirt6	Rabbit	1:1000 (1:50 for IHC, IF); Abcam, Cambridge, MA	AB62739 (Lot:GR285974)	WB,IF, IHC, Ch-IP
Sirt1	Rabbit	1:1000; Abcam, Cambridge, MA	AB28170	WB
Sirt2	Rabbit	1:1000; ProteinTech Group, Chicago, IL	19655-1-AP	WB
Sirt3	Rabbit	1:1000; ProteinTech Group, Chicago, IL	10099-1-AP	WB
Sirt4	Rabbit	1:1000; ProteinTech Group, Chicago, IL	21440-1-AP	WB
Sirt5	Rabbit	1:1000; ProteinTech Group, Chicago, IL	15122-1-AP	WB
Sirt7	Rabbit	1:1000; ProteinTech Group, Chicago, IL	12994-1-AP	WB
uPAR	Rabbit	1:1000; ProteinTech Group, Chicago, IL	10286-1-AP	WB, IF
ATG5	Rabbit	1:1000; ProteinTech Group, Chicago, IL	10181-2-AP	WB
ATG12	Rabbit	1:1000; ProteinTech Group, Chicago, IL	11122-1-AP	WB
Nephrin	Rabbit	1:1000 (1:50 for IF);Boster, Wuhan，China	BA1669	WB, IF
Podocin	Rabbit	1:1000 (1:50 for IF);Boster, Wuhan，China	BA1688	WB, IF
Notch1	Rabbit	1:800 (1:50 for IHC, IF); ProteinTech Group, Chicago, IL	10062-2-AP	WB, IF
Notch4	Rabbit	1:1000 (1:50 for IF);Abcam, Cambridge, MA	AB166605	WB, IF
H3K9ac	Rabbit	1:1000 (1:50 for IHC, IF);Abcam, Cambridge, MA	AB10812	WB, IF, IHC, Ch-IP
LC3B	Rabbit	1:1000 (1:50 for IHC); Cell Signaling, Danvers, MA	2775	WB, IHC
Beclin1	Rabbit	1:1000 (1:50 for IHC); ProteinTech Group, Chicago, IL	11306-1-AP	WB, IHC
Synaptopodin	Goat	1:100; Santa Cruz, Dallas, TX	SC-21536	IF
GAPDH	Mouse	1:4000; ProteinTech Group, Chicago, IL	60004-1-Ig	WB
